# PUF60-activated exons uncover altered 3′ splice-site selection by germline missense mutations in a single RRM

**DOI:** 10.1093/nar/gky389

**Published:** 2018-05-18

**Authors:** Jana Královičová, Ivana Ševčíková, Eva Stejskalová, Mina Obuća, Michael Hiller, David Staněk, Igor Vořechovský

**Affiliations:** 1 University of Southampton Faculty of Medicine, Southampton SO16 6YD, UK; 2 Slovak Academy of Sciences, Centre for Biosciences, 840 05 Bratislava, Slovak Republic; 3 Czech Academy of Sciences, Institute of Molecular Genetics, 142 20 Prague, Czech Republic; 4 Max Planck Institute of Molecular Cell Biology and Genetics and Max Planck Institute for the Physics of Complex Systems, Dresden, Germany

## Abstract

PUF60 is a splicing factor that binds uridine (U)-rich tracts and facilitates association of the U2 small nuclear ribonucleoprotein with primary transcripts. PUF60 deficiency (PD) causes a developmental delay coupled with intellectual disability and spinal, cardiac, ocular and renal defects, but PD pathogenesis is not understood. Using RNA-Seq, we identify human PUF60-regulated exons and show that PUF60 preferentially acts as their activator. PUF60-activated internal exons are enriched for Us upstream of their 3′ splice sites (3′ss), are preceded by longer AG dinucleotide exclusion zones and more distant branch sites, with a higher probability of unpaired interactions across a typical branch site location as compared to control exons. In contrast, PUF60-repressed exons show U-depletion with lower estimates of RNA single-strandedness. We also describe PUF60-regulated, alternatively spliced isoforms encoding other U-bound splicing factors, including PUF60 partners, suggesting that they are co-regulated in the cell, and identify PUF60-regulated exons derived from transposed elements. PD-associated amino-acid substitutions, even within a single RNA recognition motif (RRM), altered selection of competing 3′ss and branch points of a PUF60-dependent exon and the 3′ss choice was also influenced by alternative splicing of *PUF60*. Finally, we propose that differential distribution of RNA processing steps detected in cells lacking PUF60 and the PUF60-paralog RBM39 is due to the RBM39 RS domain interactions. Together, these results provide new insights into regulation of exon usage by the 3′ss organization and reveal that germline mutation heterogeneity in RRMs can enhance phenotypic variability at the level of splice-site and branch-site selection.

## INTRODUCTION

Eukaryotic genes contain intervening sequences or introns that must be removed from messenger RNA precursors (pre-mRNAs) by a large and highly dynamic RNA-protein complex, termed the spliceosome (1). Spliceosomes assemble *ad hoc* on each intron in a step-wise manner, employing U1, U2 and U4/5/6 small nuclear ribonucleoprotein particles (snRNPs) and many non-snRNP proteins (1). A critical step in the spliceosome assembly is the recruitment of U1 snRNP to 5′ splice sites (5′ss) and U2 snRNP to the branch point (BP) (1), which is facilitated by binding of the U2 auxiliary factor (U2AF) to the 3′ splice site (3′ss) (2,3). U2AF is a stable heterodimer composed of the large subunit (U2AF65), which binds U-rich sequences in the pre-mRNA, including polypyrimidine tracts (PPTs) of most annotated 3′ss (4), and the small subunit (U2AF35), which binds the 3′ss AG dinucleotide through its zinc finger domains (5). However, U-rich sequences preferentially interact with a number of other RNA-binding proteins (RBPs), including hnRNP C (6), TIA-1/TIAR (7), SRSF3 (8), PTB (9) or PUF60 (10), often in a cooperative or competitive manner. How exactly their binding regulates exon inclusion in mature transcripts on a global scale remains poorly understood.

**Figure 1.**
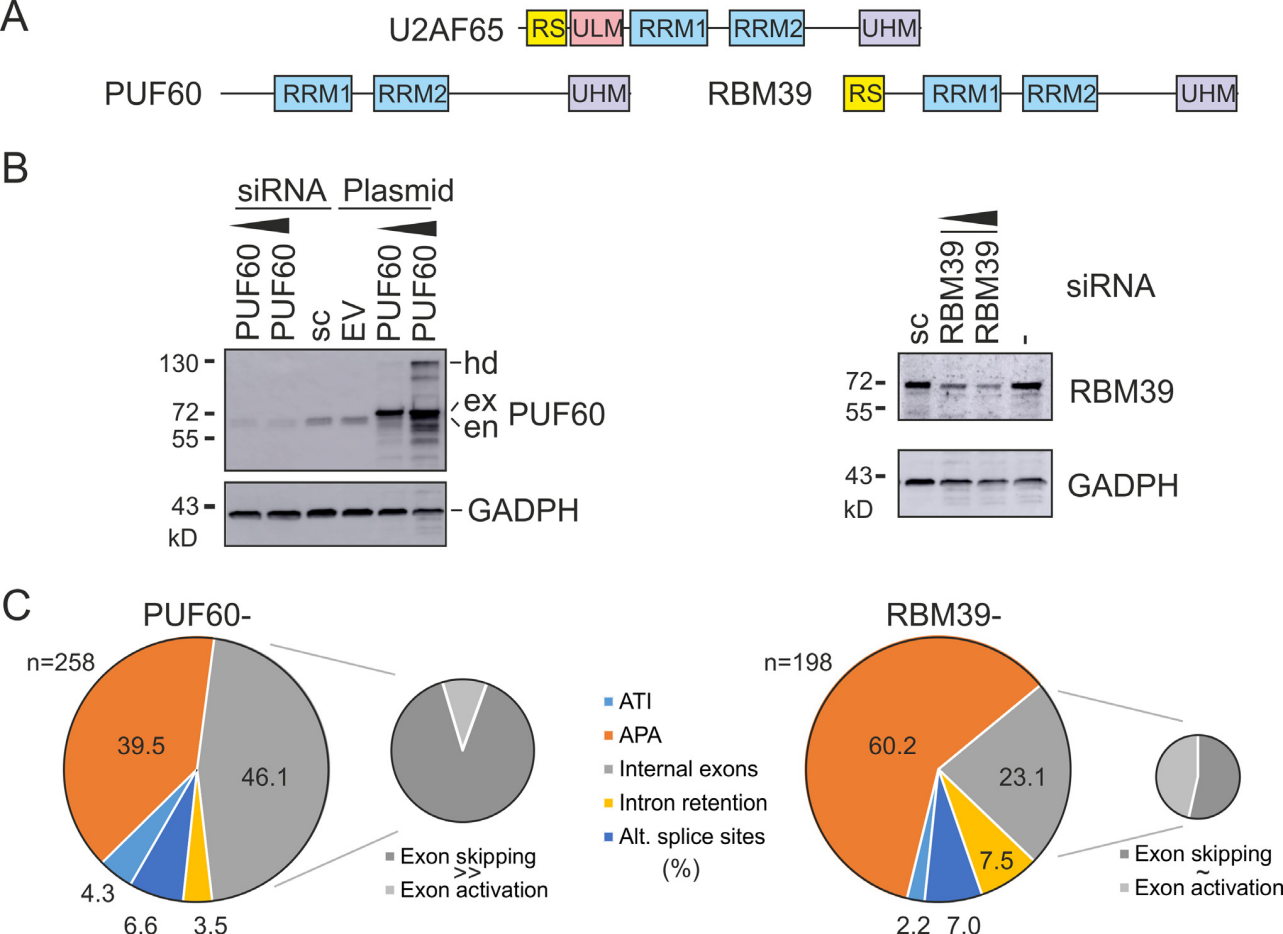
RNA-Seq of HEK293 cells depleted of PUF60 and RBM39. (**A**) Domain structure. (**B**) Western blot analysis of HEK293 cells lacking or overexpressing PUF60 (*left panel*) and lacking RBM39 (*right panel*). hd, homodimers; ex, exogenous; en, endogenous protein; sc, scrambled siRNA controls; EV, empty vector. (**C**) Distribution of RNA processing events altered by depletion of PUF60 and RBM39. Each event was confirmed in the genome browser by visualizing complete transcripts and cleaned APA sites annotated in the APA atlas (43).

PUF60 (poly-U-binding factor 60 kDa, also known as FIR, Hfp or Ro-bp1) is a splicing factor homologous to U2AF65 (10). PUF60 has two central RRMs and a C-terminal U2AF-homology motif (UHM), but lacks the N-terminal arginine/serine-rich (RS) and UHM ligand motif (ULM) domains present in U2AF65 (11,12) (Figure 1A). The PUF60-UHM does not bind nucleic acids (13) but interacts with tryptophan-containing ULMs in U2AF65, SF1 and SF3B1 (11). The PUF60-UHM and U2AF65-UHM have distinct binding preferences to ULMs at the N terminus of SF3B1 (11), a key U2 snRNP component that serves as a platform for UHM-containing spliceosome assembly factors (reviewed in 14). PUF60 activity, in conjunction with U2AF, facilitates the association of U2 snRNP with the pre-mRNA (10) and the relative abundance of PUF60 and U2AF65 can influence the choice of alternative splice sites (15). PUF60 and U2AF65 can bind SF3B1 ULMs simultaneously and noncompetitively (11), nevertheless RNA sequencing (RNA-Seq) studies revealed exons repressed by U2AF and activated by PUF60 (16), consistent with additional protein partners participating in the tight 3′ss control. Apart from the role in splicing, anti-PUF60 antibodies co-precipitated RNA polymerase II C-terminal domain and three components of the general transcription factor TFIIH, linking PUF60 to transcription (17). However, the exact function of PUF60 in global RNA processing has been unclear, despite the requirement of this protein for cell viability, proliferation and migration *in vitro* and a frequent overexpression in (pre-)malignant tissues (18,19).

RBM39 (also known as CAPERα, HCC1, FSAP59 or RNPC2) is an RNA processing factor and a hormone-dependent transcriptional co-activator with a domain structure similar to U2AF65 and PUF60 (20,21). RBM39 interacts with the U2AF/U2AF65 (22,23), SF3B1 (22,23) and RSRC1 (24). RBM39 also associated with TIA-1 and PCBP1 in a complex interacting with U2AF65 and SF3B1 to promote U2 snRNP recruitment to the BP (25). A putative *S. pombe* homolog of RBM39 (rsd1) was found to bridge U1 and U2 snRNP contacts by binding the U1 snRNP core protein U1A and Prp5 ATPase, which interacted with the SF3B1 homolog (26). RBM39 and U2AF65 share the N-terminal RS domain, which is absent in PUF60 (Figure 1A), and also RRM1/RRM2 and the C-terminal UHM (12). The U2AF65-ULM binds the RBM39-UHM with a binding affinity over four orders of magnitude weaker than binding of the U2AF65-ULM to U2AF35-UHM (27–29), yet as much as ∼20% of alternatively spliced exons appear to be co-regulated by RBM39 and U2AF65 (30). Down-regulation of RBM39 decreased the expression of cell-cycle progression regulators (31), but RBM39 function in individual RNA processing steps remains poorly understood.

Recently, independent studies have found heterozygous *PUF60* mutations in patients with a variable developmental delay, intellectual disability, spinal segmentation defects, and cardiac, ocular and renal abnormalities, first described for 8q24.3 microdeletions by Dauber *et al.* (32–36). Apart from protein-truncating *PUF60* mutations seen in most patients, missense RRM/UHM variants totalled to a third of all reported cases (32–36), suggesting that the loss of PUF60 function in PD might be conferred also by impaired RRM and UHM interactions. However, the impact of these PD alleles on RNA processing has not been examined and the development of individual PD symptoms has not been understood.

In this study, we have identified and characterized PUF60- and RBM39-dependent exons and examined functional consequences of mutations associated with human PD.

## MATERIALS AND METHODS

### Cell cultures, transfections and library preparations.

For RNA-Seq, human embryonic kidney (HEK) 293 cells were grown under standard conditions in DMEM supplemented with 10% (v/v) bovine calf serum (Life Technologies). The cells were treated with small interfering RNAs (siRNAs) targeting PUF60 or RBM39. The siRNA sequences were GCAGAUGAACUCGGUGAUGdTdT (PUF60) and GGAUCUACUGUCAUUUGUAdTdT (RBM39), as reported previously (15,37). Transfections were carried out in 6- or 12-well plates using jetPRIME (Polyplus) according to manufacturer's recommendations. The cells received the second hit after 48 h when splitting the cells into new plates, as described in detail (16). Total RNA was extracted using RNeasy Plus (Qiagen).

For library preparations, total RNA was used to isolate intact poly(A)+ RNA with the NEBNext poly(A) mRNA magnetic isolation module (E7490L), employing the Human/mouse/rat Ribo-Zero™ rRNA Removal Kit (Cambio/Epicentre) according to manufacturers’ recommendations. The libraries were prepared using the NEBNext^®^ Ultra DNA Library Prep Kit for Illumina^®^ (E7370L), size-selected and multiplexed before paired-end sequencing on the HiSeq 2500 Ultra-High-Throughput Sequencing System (Illumina) in the Wellcome Trust Centre for Human Genetics.

For transcription inhibition, HeLa cells were treated with 50 μM 5,6-dichlorobenzimidazole riboside (DRB; Sigma, D1916) for 5 h, as described (38). For co-transfection studies with PD alleles, splicing reporters (100 ng) were transfected together with wild-type (WT) and mutated PUF60 expression plasmids (200 ng) and with 30 ng of GFP plasmid DNA into HEK293 cells. We used jetPRIME (Polyplus) according to manufacturer's recommendations and isolated total RNA with the TRI reagent (Ambion).

### RNA-Seq data analysis

The raw FASTQ data were aligned against the human genome and transcriptome reference with TopHat (v. 2.0.9) (39) and Bowtie (v. 2.1.0) (40) using default stringencies and parameters, as described (41). After removal of mtRNA, rRNA, and tRNA, differential exon usage was tested using a generalized linear model implemented in a locally run DEXSeq package (v. 1.12.1) (42). Transcripts containing DEXSeq-detected exons with FDR-adjusted *P* values (*q* values) <0.05 were selected for visual verification in the Integrative Genomics Viewer (IGV) (http://www.broadinstitute.org/software/igv) to exclude false positive hits. Visualization of full transcripts was necessary to exclude errors introduced by adjacent or overlapping transcripts, misannotation of RefSeq mRNAs and low-abundance transcripts. The visual verification of DEXSeq-detected changes included inspection of alternative polyadenylation sites annotated in the APA atlas (43) in each transcript to distinguish changes in the usage of internal exons from alterations at APA sites. Only the IGV-validated events were selected for further analysis and experimental verification by RT-PCR. RNA-Seq data for PUF60 and RBM39 depletion experiments are available at ArrayExpress under the accession number E-MTAB-6010. Finally, gene- and exon-level functional enrichment analyses of differentially expressed events were performed using DAVID (44,45).

### Validation of PUF60- and RBM39-regulated exons

Total RNA from independent depletion experiments was extracted using TRI reagent, treated with DNase I (Promega) and reverse-transcribed with the Moloney murine leukemia virus reverse transcriptase (RT; Promega) and oligo(dT) primers according to the manufacturer's recommendations. PCR primers were designed to amplify two or more isoforms with different sizes (Supplementary Table S1) (16). Exogeneous transcripts were amplified using RT-PCR with vector primers PL3 and PL4 (46) or their combinations with transcript-specific primers. RT-PCR amplifications were for 28 cycles to maintain approximately linear relationship between the RNA input and signal. PCR products were extracted from the gel using GeneJET (Thermo Fisher) and sequenced to confirm the identity of each transcript. Sequencing was either direct or indirect, after subloning the gel-isolated fragments into a pGEM-T Easy vector (Promega), Signal intensities of isoforms-specific products on stained gels were measured with FluorImager using FluorQuant and Phoretix software (Nonlinear Dynamics Inc.).

### Sequence features of PUF60- and RBM39-dependent exons

Browser-validated sequences were examined using the MEME suite, including *de novo* motif discovery and motif enrichment analyses (47). Both ungapped (MEME) and gapped (GLAM2) motifs were searched. AME (47) employed known vertebrate RNA-binding motifs (48) that are relatively enriched in the input sequences compared to shuffled versions, employing background specificities. Significance of enriched motifs was tested by rank-sum tests. Prediction of BPs/PPTs and determination of AG dinucleotide exclusion zones (AGEZs) was carried out using a support vector machine (SVM) algorithm available at http://regulatorygenomics.upf.edu/Software/SVM_BP/ (49). Intrinsic splice-site strength was computed by maximum entropy modelling, a widely used scoring system useful for prediction of disease-associated aberrant 3′ and 5′ss (50,51). Alignments between short interspersed elements (SINEs) and PUF60-dependent exons were prepared by RepeatMasker, version 4.0.6, which was run in a sensitive mode with a cross-match option (v. 1.08) and the RepBase Update 20160829. Sequence logos were created using WebLogo 3 (http://www.lecb.ncifcrf.gov/∼toms/sequencelogo.html) with equiprobable background composition.

### Estimates of RNA single-strandedness

We computed PU values (Probability that an *n*-mer is Unpaired) as a measure of RNA single-strandedness for splicing regulatory sequences, employing the equilibrium partition function of RNAfold and energy minimization (52–54).The PU values were computed for IGV-validated internal exons regulated by PUF60 (*n* = 123) and control exons (*n* = 97 344). Controls were obtained from the ensGene table of the human hg38 genome assembly of the UCSC genome browser (55). Exons were restricted to internal coding exons of a single principal isoform (56); if >1 principal isoforms were present, only the longest one was selected. To ensure that their sequence context was sufficient and to minimize the impact of RNA structure signatures at splice sites (57), we extracted exons sized >60 nucleotides (nt) that have both flanking introns >400 nt. Only exons surrounded by AG..GT (*n* = 96 590) or AG..GC (*n* = 754) dinucleotides were considered. To obtain additional controls that have the same nucleotide distribution, we used *shuffle* from the *squid* package (available at http://eddylab.org/software.html) to shuffle sequences upstream/downstream of PUF60-regulated exons, excluding the splice sites and the last/first 10 nt of the intron. *Shuffle* was run with parameters -d -w and 100 to preserve both mono- and di-nucleotide distribution. Using the latest version of RNAfold (v. 2.4.3), we computed pentamer PU values as described (53,58). They were computed for three symmetrical context lengths of 10/20/30 nt up- and downstream of each pentamer. The three values were averaged and the mean was assigned to the middle pentamer position. The resulting PU values were computed for positions –100 to +10 relative to 3′ss (–1 is the G of the 3′ss AG dinucleotide, +1 is the first base of the exon) and positions –10 to +100 relative to 5′ss (–1 is the last exon base, +1 is the G of the 5′ss GT/GC dinucleotide). The PU values were averaged for each position and their means in the indicated datasets were compared by the Wilcoxon–Mann–Whitney test.

### BP mapping

Branch sites of a PUF60-dependent 3′ss of *GANAB* exon 6 and competing 3′ss of *UBE2F* exon 5 were identified using a procedure described in detail previously (59). For *GANAB*, we depleted HEK293 cells of DBR1, a debranching enzyme that cleaves the 2′-5′-phosphodiester bond at the BP (60). For *UBE2F*, HEK293 cells were co-transfected with the hybrid *UBE2F* reporter together with an empty vector or with a PUF60 expression construct that activated a competing cryptic 3′ss upstream of canonical 3′ss of exon 5. Total RNA from these cultures as well as positive and negative controls were reverse-transcribed using a gene-specific primer. Intron lariats were amplified with a set of nested primers (Supplementary Table S1). The resulting PCR products were subcloned and sequenced.

### DNA manipulations

The WT PUF60 expression construct was subcloned into BamHI/XhoI sites of pcDNA3.1/*myc-*His A (Invitrogen), employing clone pET28a-PUF60-His described previously (37), in-frame with the *myc* tag at the C-terminus. Mutations (Table 1) were created by the megaprimer overlap-extension PCR (61). PUF60 constructs expressing isoforms lacking exon 2 and/or 5 (Δ2, Δ5, Δ2Δ5) were prepared by PCR of the reverse-transcribed RNA extracted from HEK293 cells, followed by cloning into pcDNA3.1/*myc-*His A. *UBE2F* exon 5, *PUF60* exon 6 and *OGDH* exons 4a and 4b were cloned with their natural flanking intronic sequences into the *U2AF1* reporter construct (62) using XhoI/XbaI digests. *GANAB* reporters were cloned into pcDNA3.1 with EcoRI/XbaI. *PVR* reporters were cloned into pcDNA3.1/*myc-*His A using EcoRI/XbaI. Cloning primers are shown in Supplementary Table S1.

**Table 1.** PUF60 constructs expressing PD alleles

PUF60 domain	Amino acid change	Nucleotide change	Reference	Mutagenic primer^a^
RRM1	D159N	c.475G>A	(34,36)	ATCAAGAGCATCAACATGTCCTGGGA
RRM1	H169Y	c.505C>T	(32)	GTCACCATGAAGTACAAGGGCTTTG
RRM1	E181K	c.541G>A	(34,36)	TATGAGGTCCCCAAAGCTGCACAGC
UHM	V483A	c.1448T>C	(33)	TGGAAGGGGAGGCGACAGAGGAGTG
UHM	G491E	c.1472G>A	(34)	GTGGCAAGTTCGAGGCCGTGAACCG

^a^Mutations are underlined.

U1-70K-GFP-FL was cloned into pEGFP-N1 (Clontech) using BamHI/EcoRI and human U1-70K EST (38) as a template. U1-70K-CFP was generated by subcloning U1-70K-GFP-FL into pEYFP-N1 and pEYFP-C3 using EcoRI/BamHI. U1A-YFP and U1C-YFP were amplified from cDNA and inserted into pECFP-N1 using EcoRI/BamHI and XhoI/EcoRI sites, respectively. RBM39-CFP (22) was a gift from Professor Javier Cáceres, University of Edinburgh. RBM39-GFP was generated by subcloning RBM39 into pEGFP-C1 and pECFP-C1 vectors (Clontech) using BglII/BamHI. RBM39 GFP-tagged mutants were prepared by PCR, employing RBM39-GFP as a template, and subcloned into pEGFP-C1 using BglII/BamHI digests, giving rise to constructs dRS (aa 138–525), dUHM (aa 1–407) and dd (aa 138–407). Each plasmid was validated by Sanger sequencing to exclude undesired mutations.

### Immunoblotting

Western blot analyses were carried out as described (37). Antibodies against PUF60 and U2AF65 were a generous gift of Professor Adrian Krainer (Cold Spring Harbor Laboratory). Antibodies against GAPDH (SC-25778, Santa Cruz), RBM39 (PA5-31103, Thermo Fisher Scientific), U2AF35 (10334-1-AP, ProteinTech Group), TIAR (8509P, Cell Signalling Technology), TIA-1 (12133-2-AP, ProteinTech Group) and *myc* (PLA0001, Sigma) were purchased.

### Förster resonance energy transfer (FRET)

HeLa cells were transfected with fluorescent proteins-tagged constructs using Lipofectamin LTX (Invitrogen) according to the manufacturer's protocol, grown for 24–26 h and fixed in 4% paraformaldehyde/PIPES (Sigma) for 10 min at room temperature. After rinsing with PBS supplemented with Mg^2+^ and washing with water, cells were embedded in glycerol containing DABCO (Sigma). FRET efficiency was measured by the acceptor photobleaching method as described (63) using the Leica SP5 confocal microscope. Intensities of CFP (excited by the 405 nm laser set to 5–10% of the maximum power) and YFP (excited by the 514 nm laser set to 2% of the maximum power) were measured and YFP was then bleached in the region of interest by three intensive (30% maximum power) pulses of a 514 nm laser line, followed by repeated CFP and YFP fluorescence measurements. The FRET efficiency (%) was measured in three independent experiments, each containing at least 10 cells. The FRET efficiency was calculated as (CFP _after_ – CFP _before_) × 100/CFP _after_.

### Immunoprecipitation (IP)

HeLa cells were grown on 15 cm Petri dishes to ∼60% confluence and then transfected with 1 μg of plasmid DNA per 1 ml of DMEM using Lipofectamine LTX (Invitrogen) according to manufacturer's instructions. Cells were harvested at >90% confluency into buffer NET2 (50 mM Tris–HCl pH 7.5, 150 mM NaCl, 0.05% Nonidet P-40) supplemented with a complete mix of protease inhibitors (Calbiochem) and pulse-sonicated on ice for 90 s. Cell extracts were incubated (2 h) with Protein-G agarose beads (GE Healthcare) covered with goat polyclonal antibodies against GFP (a generous gift from Dr David Drechsel, MPI-CBG, Dresden, Germany). Co-immunoprecipitated proteins were eluted into the protein sample buffer (4% SDS, 20% glycerol, 10% 2-mercaptoethanol, 0.004% bromophenol blue, 0.125 M Tris–HCl, pH ∼6.8) and incubated at 95°C for 10 min. Proteins were resolved on SDS-PAGE, transferred onto membranes and probed with anti-GFP (mouse monoclonal, clone B-2, Santa Cruz), anti-RBM39 (rabbit polyclonal, HPA001591 Sigma), anti-U2AF35 (rabbit polyclonal, AB86305, Abcam), anti-U1-70K (rabbit polyclonal AV40276, Sigma), anti-U1C (rabbit monoclonal, ab192028, Abcam) and anti-SF3B4 (mouse monoclonal, ab104226, Abcam) antibodies.

## RESULTS

### Identification of human exons regulated by PUF60 or RBM39

RNA-Seq of HEK293 cells individually depleted of PUF60 or RBM39 (Figure 1A,B) followed by the DEXSeq analysis of exon usage identified a total of 689 up- or down-regulated events in PUF60-depleted (PUF60-) cells and 376 up- or down-regulated events in RBM39-depleted (RBM39-) cells. Visualization of each exon with log_2_fold values >0.3 (1.23×) and >0.1 (1.07×) in the context of full transcripts in IGV confirmed 258 events in PUF60- cells and 198 events in RBM39- cells, respectively (Supplementary Table S2). *PUF60* transcripts in PUF60- cells were reduced ∼10-fold (Supplementary Table S3) and lacked an alternatively spliced exon 5 (Supplementary Figure S1A), confirming the previous observation (32). *RBM39* transcripts in RBM39- cells were reduced less and had no exon usage alterations (Figure 1B, Supplementary Table S3). In PUF60- cells, we observed *U2AF1* upregulation and *U2AF2* downregulation at the transcript level and exon skipping in *RBM23* (*CAPERβ*) whereas *RBM39* was slightly upregulated (Supplementary Figure S1B–D). Supplementary Table S3 gives a summary of gene-level alterations found for UHM- and ULM-encoding transcripts in PUF60- and RBM39- cells.

Distribution of individual RNA processing steps affected by PUF60 and RBM39 depletion is shown in Figure 1C and their functional examples in Supplementary Figure S1E–G. As compared to RBM39- cells, PUF60- cells exhibited a larger proportion of differentially used internal exons with no apparent link to altered usage of annotated alternative polyadenylation (APA) sites (Figure 1C). The fraction of APA changes was significantly higher in RBM39- cells than in PUF60- cells (*P*<.0001, χ^2^ = 17.1), despite the more efficient PUF60 depletion (Figure 1B, Supplementary Table S3). Whereas skipping and activation of internal exons was about equally represented in RBM39- cells, their distribution was significantly biased towards exon skipping in PUF60- cells (Figure 1C, insets), indicating that PUF60 acts mainly as a splicing activator. Collectively, these results revealed a large set of human internal exons skipped in PUF60- cells (termed PUF60-activated exons) and suggested a distinct function of PUF60 and RBM39 in the two RNA processing steps.

### Sequence features of PUF60-activated and -repressed exons

**Figure 2.**
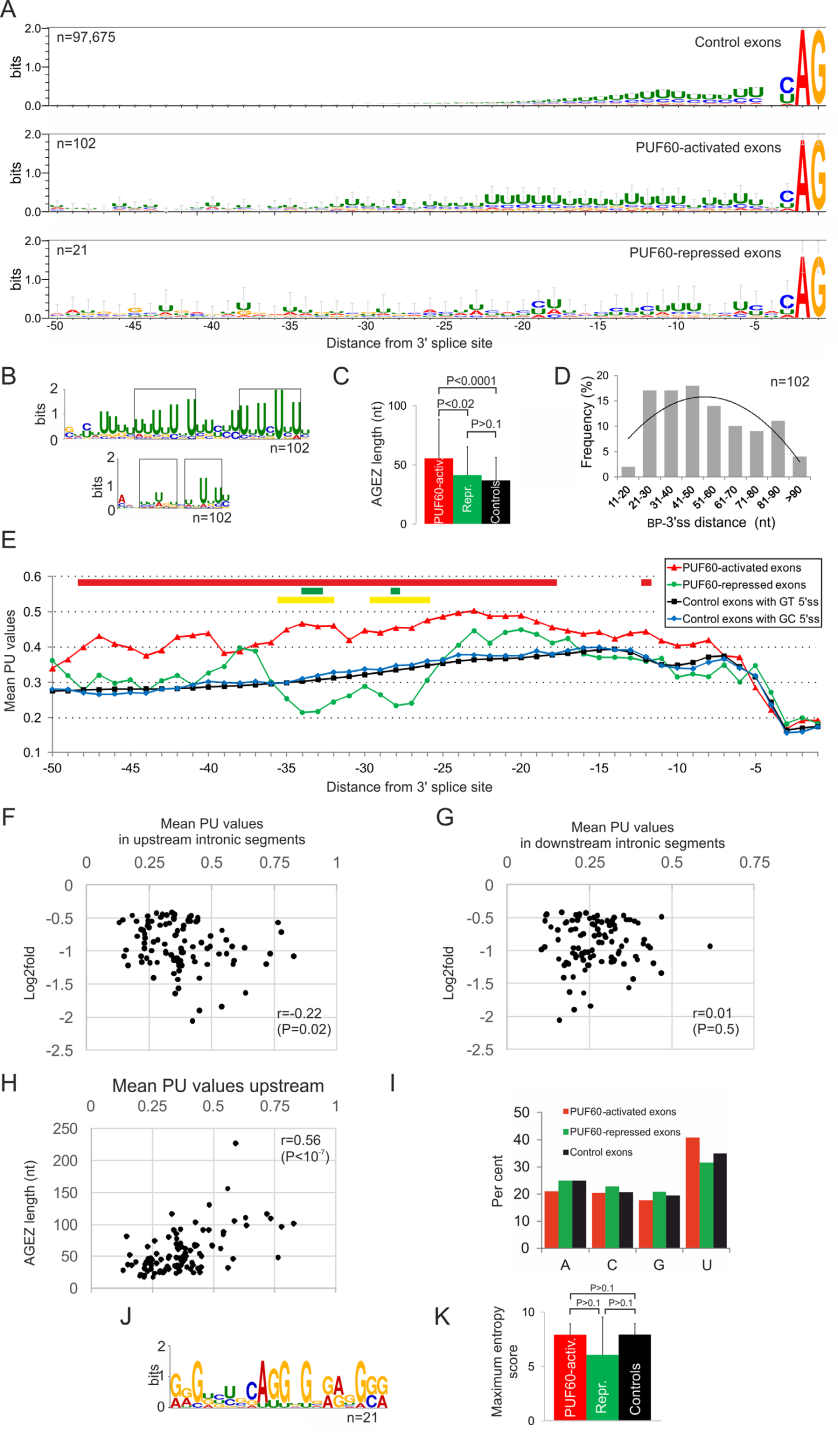
Sequence characteristics of PUF60-regulated exons. (**A**) Information content upstream of PUF60-activated and -repressed exons and controls. The overall height of the stack shows the relative frequency of the indicated nucleotides at each position. Error bars display Bayesian 95% confidence intervals. Number of exons is to the left. (**B**) *De novo* motif discovery upstream of PUF60-activated exons with MEME. The *upper panel* shows a motif with the lowest *E* value (2.1e^−28^) for input sequences between positions –100 and –4 relative to 3′ss; the *lower panel* shows a motif with the lowest *E* value for input sequences between –50 and –18. Rectangles denote two subregions with alternating uridines. (**C**) AGEZ length of PUF60-regulated and control exons. Error bars, SD. *P*-values were derived by the Wilcoxon–Mann–Whitney test. (**D**) Frequency distribution of BP-to-3′ss distances predicted for PUF60-activated exons. (**E**) Mean PU values, estimating RNA singlestrandedness, upstream of PUF60-regulated and control exons. Coloured rectangles at the top denote significant intron positions when comparing the means of PUF60-activated exons and controls (red; *P*-values <0.01), PUF60-repressed exons and controls (green, *P*-values <0.1) and PUF60-activated and -repressed exons (yellow, *P*-values <0.05). (F, G) Correlation of the mean PU values upstream (**F**) or downstream (**G**) of 102 PUF60-activated 3′ss with their log_2_fold values. The PU means were computed for positions –4 to –100 and +7 to +100 upstream and downstream of each exon, respectively. (**H**) The AGEZ length correlates with the predicted RNA singlestrandedness upstream of PUF60-activated 3′ss. (**I**) Mean nucleobase frequencies in 100-nt intronic flanks upstream of PUF60-regulated exons. (**J**). *De novo* motif identified upstream of PUF60-repressed exons (*E*-value: 3.6e^−4^). (**K**) Mean maximum entropy scores (50) for 3′ss of PUF60-regulated exons and controls. Error bars, SD.

Sequence analysis of 102 PUF60-activated internal exons that were not associated with APA (Supplementary Table S2) showed that upstream intronic sequences flanking 3′ss were enriched for uridines (U) as compared to control exons (Figure 2A). The enrichment was most pronounced between ∼18 to ∼50 nt upstream, but was absent across these exons or downstream of their 5′ss (Figure 2A, Supplementary Figure S2A,B, and data not shown). The most significant *de novo* motif identified by MEME in sequences upstream of PUF60-activated 3′ss revealed two U-rich subregions, each with alternating U frequencies, that were separated by a short U-rich segment without this pattern (Figure 2B, *upper panel* and Supplementary Table S4). The average distance between the motif start position and the 3′ss was 48 nt. The bipartite pattern was much reduced but not completely eliminated when the input was limited to positions –18 to –50 (Figure 2B, *lower panel*). Motif enrichment analysis against the compendium of known RBPs (48) by AME revealed the most significant enrichment for UGUGUGU (Supplementary Table S5), a motif bound also by Bruno-like proteins (also known as CUGBP-Elva-like family, or CELF) and *Drosophila* PAPI. Alternative UG 3′ss previously identified in the vicinity of AG 3′ss (64) were not detected by DEXSeq. *De novo* motif analysis of downstream introns revealed significant signatures containing GGC repeats and adenine stretches (Supplementary Figure S2C and D), which might facilitate exon looping via U-A base pairing and spatial approximation of 3′ss and 5′ss.

3′ss are preceded by variable regions devoid of AG dinucleotides known as AG dinucleotide exclusion zones (AGEZs), which contribute to accurate selection of 3′ss (65). We found that the mean AGEZ was 1.51x longer for PUF60-activated exons than for controls (Figure 2C). These exons also showed a significant increase in the mean distance between BPs with the highest SVM score and 3′ss (∼52 nt), as opposed to the average of ∼25 nt (Figure 2D) previously estimated (49) or determined by RNA-Seq (66) for human exons.

The U-rich regions of PUF60-activated 3′ss were mirrored by an increased RNA single-strandedness, with the peak of PU values shifted further upstream of 3′ss as compared to human control exons, having either GT or GC 5′ss (Figure 2E and Supplementary Figure S3). GC 5′ss, which are present in ∼1% of human exons, showed an enhanced single-strandedness at positions –4 to –7 relative to the exon–intron junction as compared to GT 5′ss, consistent with their requirement for splicing enhancers in this region (67). Intron positions +3 through +7 also exhibited significant differences in mean PU values when comparing GT 5′ss with GC 5′ss (Supplementary Figure S3, *inset*). The PU values averaged for positions –18 to –40 nt upstream of PUF60-activated 3′ss, a typical location of human BPs, were significantly higher than for control exons (0.453 versus 0.330, *P* < 0.01). We also detected an increase in PU values 6–8 nt upstream of their 5′ss. The PU values computed for 100-nt flanking intronic segments upstream but not downstream of PUF60-activated exons correlated with changes in exon inclusion levels in PUF60- cells (Figure 2F and G). Finally, the AGEZ length strongly correlated with PU values upstream (Figure 2H) but not downstream (*r* = –0.02) of PUF60-activated exons.

Because the number of internal exons upregulated in PUF60- cells and not associated with altered APA sites (termed PUF60-repressed exons) was low for a comparable analyses (Figure 1C), we extended the sample to include exons beyond the DEXSeq threshold value, revealing a total of 21 IGV-validated events (Supplementary Table S2). Despite the small number, these exons showed U depletion upstream of 3′ss (Figure 2A and I), which was accompanied by a reduction in predicted single-strandedness (Figure 2E). The most significant purine-rich *de novo* motif identified in their high-guanine background is shown in Figure 2J. The AGEZ length of PUF60-repressed exons was similar to controls (Figure 2C) as was the mean distance (∼24 nt) between predicted BPs and 3′ss. Finally, the intrinsic strength of 3′ss, as estimated by maximum entropy scores, was similar in each exon group (Figure 2K).

The number of IGV-validated RBM39-dependent and APA-unrelated internal exons was much lower than for PUF60, precluding similar analyses. However, exons downregulated in RBM39- cells also displayed U-rich regions upstream of 3′ss (Supplementary Figure S4), with two exons found both in PUF60- and RBM39-activated sets (in *DLGAP5* and *PIGP*, Supplementary Table S2). An example of an APA site co-regulated by the two proteins is shown in Supplementary Figure S1F–G.

Taken together, global sequence characteristics of PUF60-activated exons are consistent with a requirement for functionally significant PUF60 binding to more accessible pre-mRNA segments upstream of a large subset of 3′ss to promote their use.

### 3′ss organization and concordant and discordant usage of internal exons by PUF60 and U2AF

**Figure 3.**
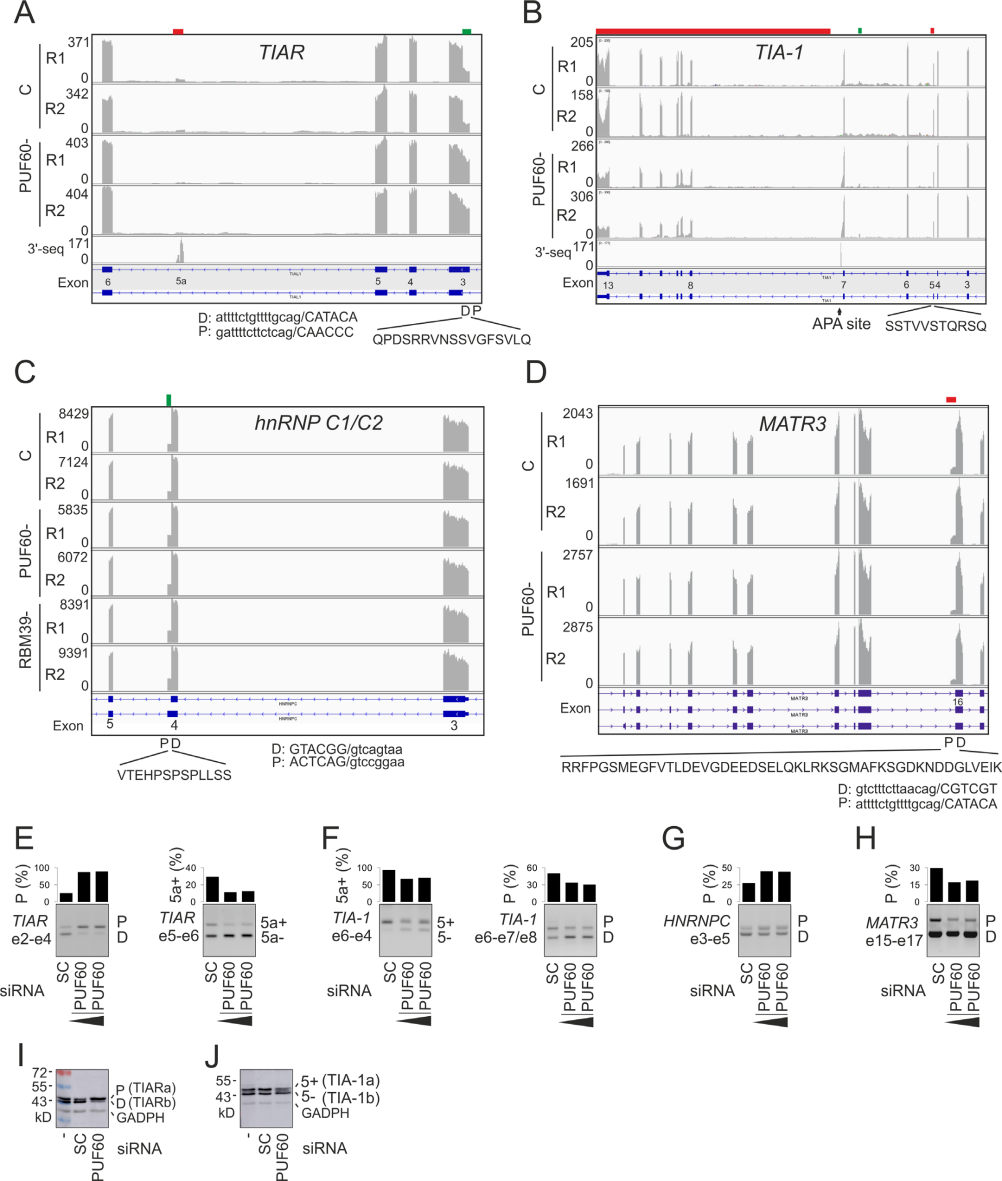
Alternative splicing of U-binding interaction partners of PUF60 in depleted cells. **(A–D)** Genome browser views of RNA-Seq tracks in control (C) and depleted (–) cells. Down- and up-regulated exonic segments are marked by red and green rectangles at the top, respectively. Y-axis, sequencing read numbers. R1, R2; replicates. Peptides encoded by PUF60/RBM39-dependent exons are shown at the bottom together with their intron-proximal (P) or -distal (D) 3′ss. The 3′-seq tracts superimpose the APA atlas data (43). (**A**) *TIAR*. (**B**) *TIA-1*. (**C**) *HNRNPC*. (**D**) *MATR3/SNHG4*. (**E–H**) RT-PCR validation from independent transfections. The final siRNA concentrations were 50 and 90 nM. SC, scrambled controls. Exons (e) containing amplification primers (Supplementary Table S1) are to the left and RNA products are to the right in each panel. Columns show the relative abundance of the indicated transcripts (shown in panels A–D). (**I, J**) Immunoblotting of PUF60- and control cells with anti-TIAR **(I)** and anti-TIA-1 **(J)** antibodies. The extra band between TIA-1a and TIA-1b is likely to result from phosphorylated residue(s) reported in the peptide shown in panel B (http://www.phosphosite.org).

Comparison of U2AF(35)-dependent (16) and PUF60-regulated internal exons revealed many examples of PUF60-activated exons and U2AF(35)-repressed exons (Supplementary Table S2 and Supplementary Figure S5). The two groups of exons shared extended PPTs and the BP-3′ss distance, the increased AGEZ length and also increased PU values upstream of 3′ss (cf. Figure 3J in (16) with Figure 2E). In contrast, these sequence characteristics were observed neither for PUF60-repressed and U2AF(35)-activated exons, nor were they apparent for concordant exons. We illustrate this contrasting 3′ss organization in the context of adjacent, duplicated exons (Supplementary Figure S6). *OGDH* exon 4a, which is preceded by a weak PPT and intron-distal BPs, was repressed by PUF60 and activated by U2AF subunits; by contrast, *OGDH* exon 4b was activated by PUF60 and repressed by U2AF. Exon 4b is preceded by a long U-rich PPT and a high-score distant BP(s) near the 5′ss of intron 4a. The predicted BP(s) are close to or below a minimum distance threshold (∼50 nt) for the productive U1-U2 assembly (68), most likely enforcing the mutually exclusive splicing of exons 4a and 4b. This U2AF/PUF60 regulation is functionally important: transcripts containing exon 4b encode a Ca^2+^-sensitive variant of the E1 subunit of the 2-oxoglutarate dehydrogenase complex and are expressed at >95% in the heart and skeletal muscles, whereas transcripts containing exon 4a code for a Ca^2+^-insensitive counterpart, which shows a high (∼50%) expression in the brain (69).

### PUF60- and RBM39-regulated exon usage in genes encoding their interaction partners

Figure 3A–J shows exon-centric co-regulation of protein interaction partners of PUF60 that contact U-rich motifs. The lack of PUF60 was associated with activation of intron-proximal 3′ss of *TIAR (TIAL1)* exon 3 and the inclusion of its 51-nt portion into the mRNA. This segment encodes an extra 17 residues within the RRM1 domain (Figure 3A,E,I). Unlike TIAR RRM2 or RRM3, the RRM1 domain does not strongly interact with cellular RNA (7,70) and may potentially function as UHM in the TIAR paralog TIA-1 (70). Apart from the PUF60-dependent alternative 3′ss usage, we observed repression of a cryptic *TIAR* exon at an annotated APA site (Figure 3A and E), which introduces a stop codon. Thus, these PUF60-regulated events are likely to control *TIAR* levels and/or protein interactions of TIAR rather than RNA binding.

In *TIA-1*, PUF60 depletion activated the annotated intronic APA site by ∼2-fold, and reduced inclusion levels of alternative exon 5 (Figure 3B, F and J). The former change would be predicted to limit interactions between the C-terminal domain of TIA-1 and its ligands whereas the latter would increase the ratio of TIA-1a/TIA-1b isoforms. The two isoforms differ by 11 aa, but show similar subcellular distribution and RNA binding, although the shorter TIA-1b displayed an enhanced splicing stimulatory activity compared with TIA-1a, both *in vitro* and *in vivo* (71). Thus, a lack of PUF60 alters the expression of TIA-1 and TIAR isoforms at the level of pre-mRNA splicing and protein expression.

TIAR interacts with hnRNP C1/C2 and MATR3 (72) and both proteins bind U-rich motifs (6,48). The hnRNP C1/C2 interaction is mediated by the Q-rich C-terminal domain of TIAR and C-terminal domains of hnRNP C1/C2 isoforms (72). hnRNP C1 and C2 are produced by alternative 5′ss of exon 4 that are separated by 39 nt, which code for a low-complexity peptide containing phosphorylated serines (73). The intron-proximal 5′ss was promoted in PUF60- cells, increasing the relative abundance of the longer hnRNP C2 (Figure 3C and G). Thus, the availability of the phosphorylated hnRNP C2 isoform is responsive to concentrations of U-binding interaction partners through alternative 5′ss control.

Apart from altering 3′ss selection of a *SNHG4* exon, potentially influencing chimeric *SNHG4-MATR3* transcripts (74), PUF60- cells showed repression of an intron-proximal 3′ss of *MATR3* exon 16 (Figure 3D and H), reducing the relative expression of mRNAs containing extra 144 nt. This segment inserts 48 amino-acids in this UC-binding protein, close to the nuclear localization signal encoded by the preceding exon. Finally, we observed no changes in *RALY, TARDBP* and *ELAVL1* (HuR) transcripts. Overall, these results reveal a PUF60-dependent regulation of U-binding proteins that interact with each other.

### Co-regulation of U-binding hnRNPs by PUF60/RBM39

**Figure 4.**
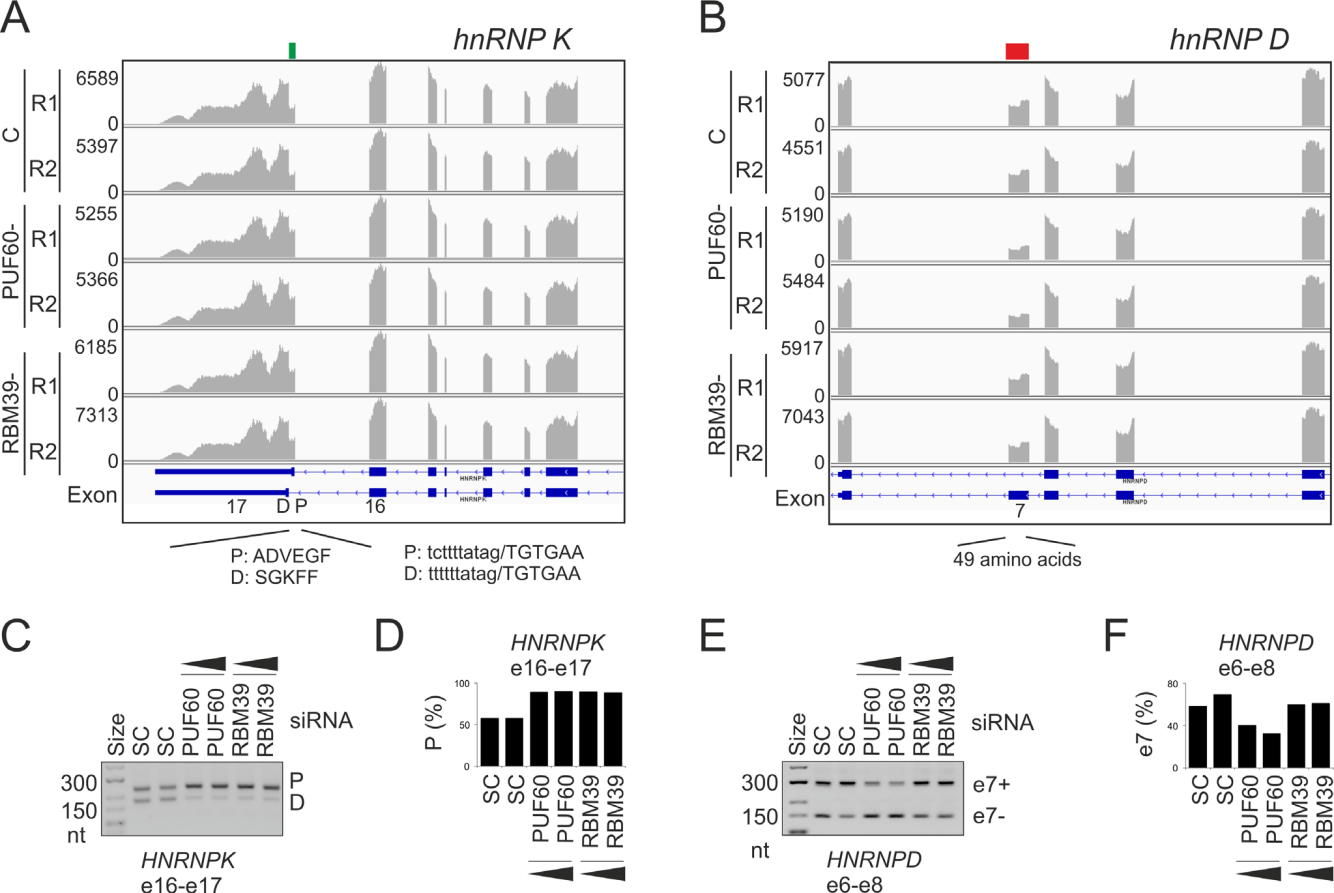
Alternative splicing of hnRNP genes regulated by PUF60/RBM39. (**A**) *HNRNPK*. (**B**) *HNRNPD*. (**C–F**) RT-PCR validation. Down- and up-regulated exonic segments are marked by red and green rectangles at the top, respectively. The siRNA concentrations were 50 and 90 nM. SC, scrambled siRNA controls. Exons (e) targeted by amplification primers (Supplementary Table S1) are at the bottom. Columns show the relative abundance of the indicated transcripts (shown in panels A and B).

PUF60 or RBM39 depletion also influenced alternative splicing of other hnRNPs that bind U-rich motifs (Figure 4A–F). Both PUF60 and RBM39 repressed the proximal 3′ss of the last hnRNP K exon (Figure 4A, C and D). hnRNP K, which binds UC-rich stretches but lacks RRMs (75), exists in two isoforms that differ at their C termini as a result of alternative 3′ss. The isoform employing the proximal 3′ss preferentially accumulates in the cytoplasm as compared to distal 3′ss (76). The proximal 3′ss has a longer upstream U-rich region, consistent with a higher affinity of PUF60/RBM39 to the longer PPT.

hnRNP D (AUF1) isoforms are produced by alternative splicing of exons 2 and 7 (77). Exon 7 requires PUF60 (Figure 4B, E and F); it encodes a long peptide at the C-terminus in two of the four hnRNP D isoforms. All isoforms shuttle between nucleus and cytoplasm in a transcription-independent manner (78). Nuclear import signal is located in the C-terminal domain of only smaller hnRNP D isoforms (p40 and p37) whereas nuclear export is facilitated by an extra peptide encoded by exon 7, which is included in larger isoforms (p45 and p42) (78). The extra peptide inhibits ubiquitination and rapid degradation of hnRNP D (79) and is also required for interaction with tristetraprolin, increasing its binding to AU-rich elements (80). Thus, physiological PUF60 levels are important for nuclear export of hnRNP D through alternative splicing of *HNRNPD* exon 7.

Finally, the gene encoding hnRNP M, which interacts with PUF60, generates at least two protein isoforms, with isoform M1 lacking a 39-aa peptide encoded by the retained intron 6 (81). Retention of this intron was repressed in PUF60- cells (Supplementary Figure S7). Interestingly, hnRNP M binds poly(U) and poly(G) homopolymers and UG-rich sequences that often contain UU dinucleotides (82,83). The extra peptide is inserted between hnRNP M RRM1 and RRM2, possibly influencing RNA binding.

Taken together, these results uncover co-regulation of U-bound hnRNPs by PUF60 and/or RBM39 and identify alternative RNA processing events that reveal their coordinated expression, linking the two UHM-containing proteins to mainstream splicing regulators.

### PUF60 activates antisense SINE exons

Short interspersed elements (SINEs) are the most abundant transposed elements in the human genome, with >1.5 million copies of mammalian-wide interspersed repeats (MIRs) and younger, primate-specific *Alu*s (84). Inclusion of SINE-derived exons in mRNAs is regulated by U-binding proteins, including hnRNP C and U2AF65 at the 3′ss/PPT (6), and TIA proteins at the 5′ss (85). To test if PUF60 contributes to this regulation, we examined PUF60-activated and -repressed exons for the presence of transposed elements. SINE exons were present in each exon group (Supplementary Figures S8 and S9). In PUF60-activated exons, *Alu* and MIR fragments were mostly in antisense orientation, with frequent T>G substitutions in the poly(A) tails of ancient free left *Alu* monomers (FLAMs) (Supplementary Figure S8A). FLAMs predate dimeric *Alu*s and are similar to rodent B1 elements (86). Both major FLAM subfamilies (A and C) were represented and their reduced exon inclusion in PUF60- cells was validated by RT-PCR (Supplementary Figure S8B). A search of the Transpogene database (87) for exonized *Alu*s with [(T)_n_G]_n_ repeats in their PPTs identified 40 candidate exons (Supplementary Table S6), which had a higher and lower representation of old *Alu*J and young *Alu*Y subfamilies, respectively (Supplementary Figure S8C). Browser inspection of these exons revealed an additional PUF60-activated exon not detected by stringent DEXSeq criteria (Supplementary Figure S8D), but most of these exons were not expressed in our cells at all. In contrast, transposons found in PUF60-repressed exons were mostly in the sense orientation, with poly(A) tails of *Alu* fragments located further away from splice sites (Supplementary Figure S9). Taken together, we identified PUF60-regulated SINE exons whose activation appears to be facilitated by more diverged PPTs of antisense *Alu* copies.

### Functional annotation of genes with PUF60-dependent RNA processing events

Functional annotation clustering of genes with PUF60-dependent exons using DAVID (45) showed a significant enrichment for FERM (F for 4.1 proteins, E for ezrin, R for radixin and M for moesin) domains (Supplementary Figure S10), which are found in numerous proteins at the interface between the membrane and the cytoskeleton. The enrichment for cytoskeleton-encoding genes including actin/spectrin-associated factors was observed also for U2AF35 (16), raising a hypothesis that the two factors or perhaps other UHM-containing proteins have been important for the association of cytoskeletal proteins to cytoplasmic tails of integral membrane proteins. Genes with PUF60-dependent exons lacked zinc finger-binding domains, in contrast to U2AF(35)-dependent exons (16).

### Association of RBM39 with U1 snRNP via U1-70K

**Figure 5.**
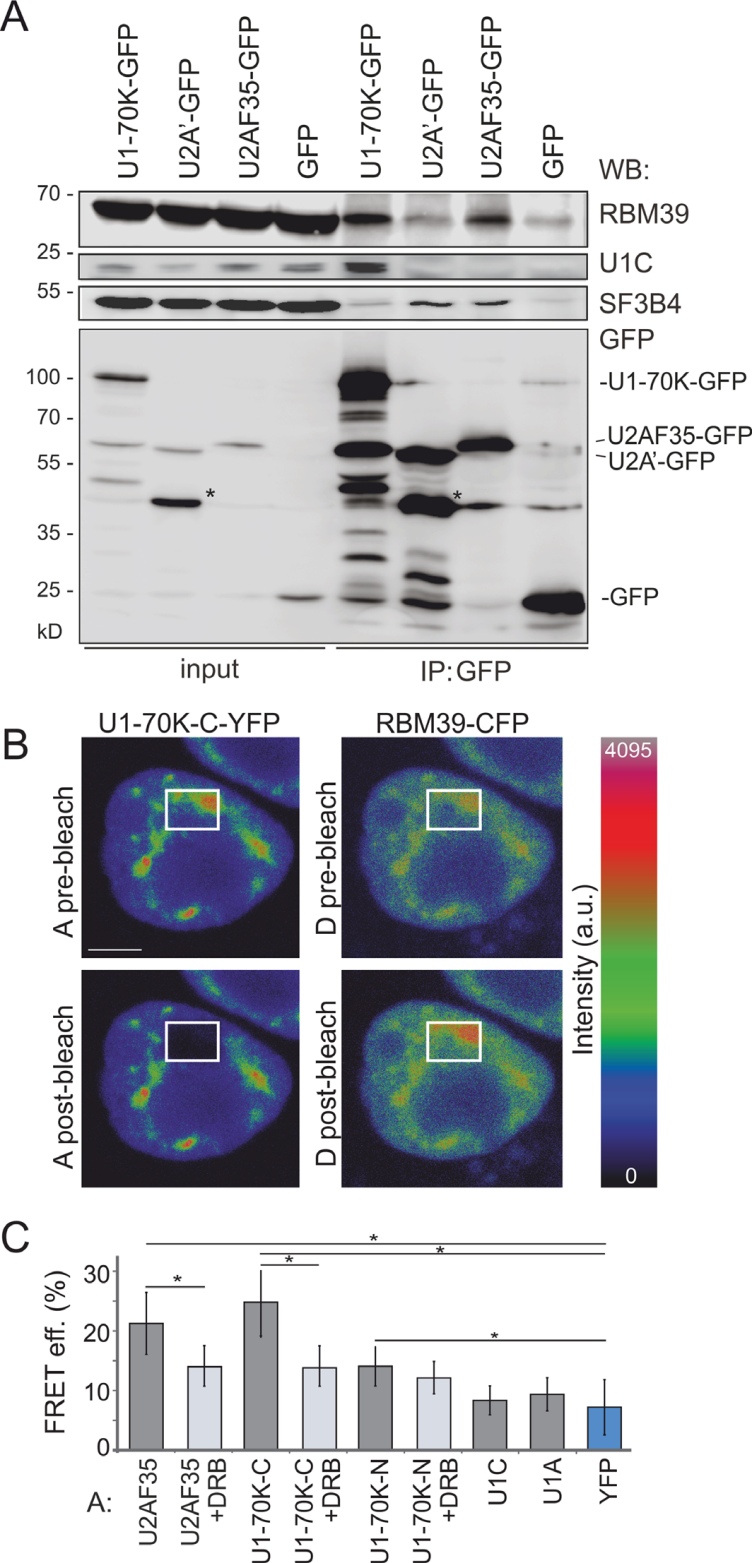
RBM39 interactions with spliceosome components. (**A**) RBM39 interacts with U1 snRNP and U2AF. Interaction of RBM39 with the U1-specific protein U1-70K, the U2-specific protein U2A’ and the small subunit of U2AF was assayed by immunoprecipitations. HeLa cells were transiently transfected with U1-70K-GFP, U2A′-GFP or U2AF35-GFP, immunoprecipitated with anti-GFP antibodies and probed with antibodies shown to the right. U1C and SF3B4 served as positive controls for immunoprecipitations for U1-70K-GFP and U2A’-GFP, respectively. Asterisks denote a partially degraded U2A’-GFP. **(B, C)** RBM39 interactions monitored by FRET. Cells were transiently co-transfected with RBM39-CFP and C-terminally YFP-tagged U1-70K. (**B**) YFP was bleached in a small region comprising the nucleoplasm and nuclear speckles; CFP fluorescence was measured before and after bleaching. Fluorescence of RBM39 increased after bleaching of U1-70K-YFP [cf. CFP fluorescence in the bleached region (rectangles) before (*top panel*) and after (*bottom panel*) bleaching]. A, acceptor; D, donor; scale bar, 5 μm. (**C**) Quantification of individual donor-acceptor FRET efficiencies upon the inhibition of RNA polymerase II by DRB. Columns indicate means; errors bars SEMs. Interaction between RBM39-CFP and U2AF35-YFP (22) served as a positive control and interaction between RBM39-CFP and YFP as a negative control. Significantly different means are denoted by an asterisk (*P*< 0.01; *t*-test).

Unlike U2AF65, RBM39-RNA interactions were observed close to transcription start/termination sites in 5′ and 3′ untranslated regions (30). To explore if the observed APA preference of RBM39 could be mediated by the N-terminal RS domain, which is absent in PUF60 (Figure 1A and C), we first transiently expressed GFP-tagged U1 and U2 snRNP components (U1-70K and U2A’) and the U2 recruitment factor U2AF35 in HeLa cells and assayed for interactions with endogenous RBM39. Endogenous RBM39 was pulled down by U1-70K and U2AF35 but not by U2A’ (Figure 5A), suggesting that RBM39 interacts with U1 snRNP constituents and U2AF. To confirm these interactions, we co-expressed the RBM39-CFP construct with YFP-tagged U2AF35, U1-70K, U1C and U1A and examined their association by FRET assayed by the acceptor photobleaching method (Figure 5B). The FRET assay confirmed the association of RBM39 with U2AF35 (22) and showed that RBM39 interacted preferentially with U1-70K as the FRET efficiency of RBM39-GFP with other U1-specific proteins U1C-YFP and U1A-YFP was not above the background levels. The RBM39 interaction with U1-70K and U2AF35 was transcription-dependent since the inhibition of RNA polymerase II activity with DRB reduced the FRET signal between donor RBM39-CFP and acceptors U2F35-YFP or U1-70K-YFP (Figure 5C).

**Figure 6.**
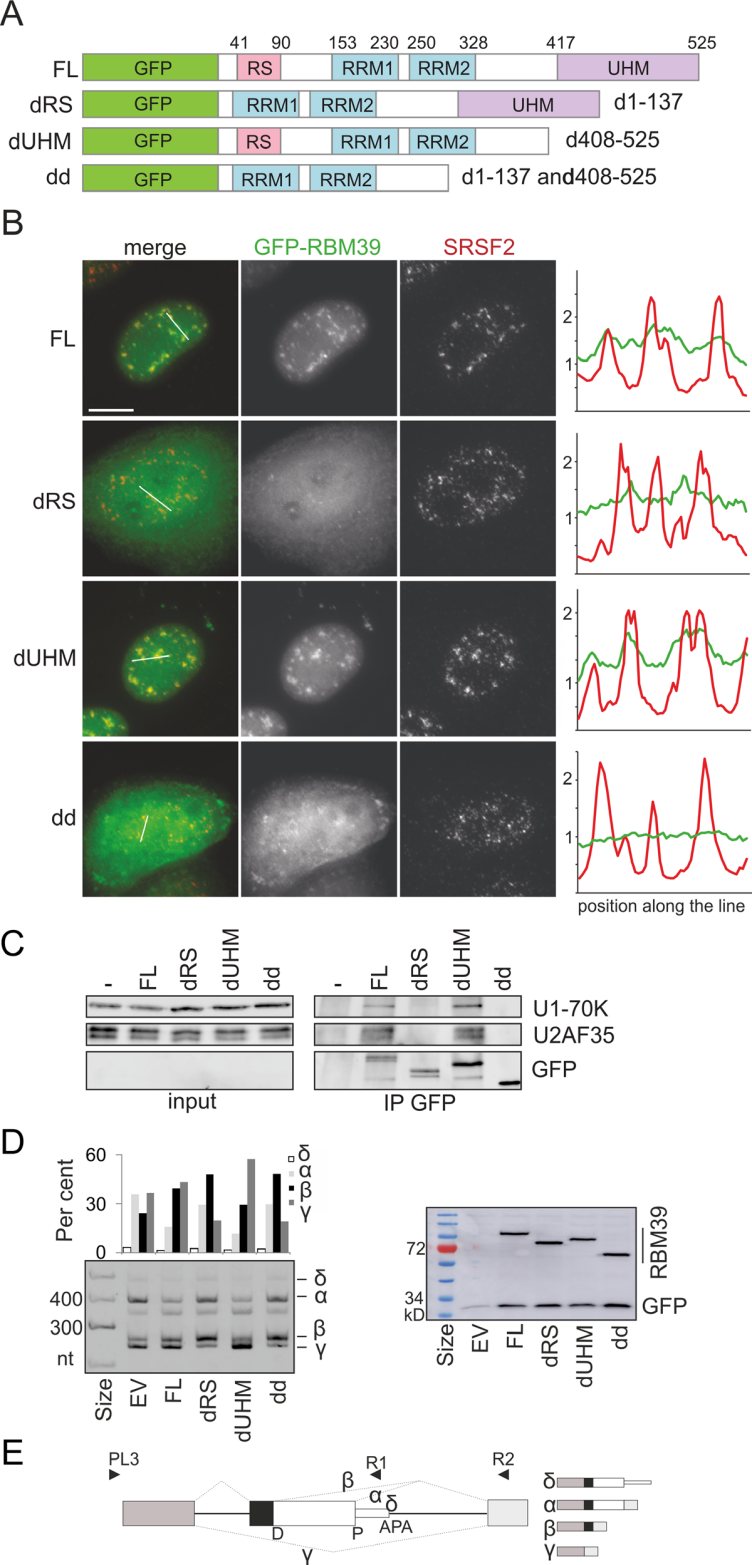
RBM39 RS domain is responsible for nuclear localization and interactions with U1-70K and U2AF35. (**A**) RBM39 domains deletion (d) constructs. FL, full-length protein; dd, double deletion. Deleted amino acids are to the right. (**B**) The RS domain of RBM39 is important for localization into nuclear speckles. HeLa cells expressing GFP-tagged RBM39 constructs (green) were immunostained with the anti-SRSF2 antibody (red), which marks nuclear speckles. Scale bar, 10 μm. *y*-axis, fluorescence intensity (arbitrary units ×10^3^). (**C**) The N-terminal segment with the RS domain is responsible for interaction with U1-70K and U2AF35. Transiently transfected GFP-tagged RBM39 mutants were immunoprecipitated using anti-GFP antibodies and co-precipitated proteins were visualized by western blotting. Non-transfected HeLa cells served as a negative control. (**D**) Isoform expression of exogenous poliovirus receptor (*PVR*) transcripts (*left panel*) in cells transiently co-transfected with RBM39 deletion constructs and GFP plasmids as transfection/loading controls (*right panel*). The membrane was incubated with anti-GFP antibodies. EV, empty vector. (**E**) *PVR* minigene schematics. D,P, distal and proximal 5′ss; arrowheads, PCR primers (Supplementary Table S1); dotted lines, PVR isoforms (schematically shown to the right).

To test which RBM39 domain is required for the interactions with U1-70K and U2AF35, we examined GFP-tagged UHM- and RS-deletion constructs transiently expressed in HeLa cells (Figure 6A and B). The full-length protein as well as the protein lacking the UHM domain co-localized with SRSF2 in nuclear speckles. In contrast, mutants without the RS or RS/UHM domains showed diffused nucleo-cytoplasmic localization, indicating that the first 137 aa containing the RS domain are essential for targeting RBM39 into the nucleus and nuclear speckles. Mutated proteins lacking this segment were also unable to co-precipitate U1-70K or U2AF35 (Figure 6C). Finally, deletion constructs with impaired cellular localization and U1-70K/U2AF35 contacts also failed to restore RBM39-dependent RNA processing of an exogeneously expressed reporter derived from the poliovirus receptor (*PVR)*, namely, reduce *PVRα* isoforms by repressing a proximal splice site (Figure 6D and E). Constructs lacking the RS domain promoted the *PVRβ* isoform, suggesting that the RS domain is important for recognition of the proximal splice site and/or repression of the distal site. Thus, the remaining nuclear dRS pool can still associate with other factors or RNA and influence splicing. In contrast, the construct lacking UHM was proficient, but induced more *PVRγ* than the WT RBM39, indicating that the UHM domain is required for inclusion of the APA exon. Together, these results show that RBM39 interacts with U1-70K and U2AF via the N-terminal part incorporating the RS domain and identify RBM39 domain-dependent RNA processing events.

### PD-associated missense mutations in a single RRM select distinct 3′ss

**Figure 7.**
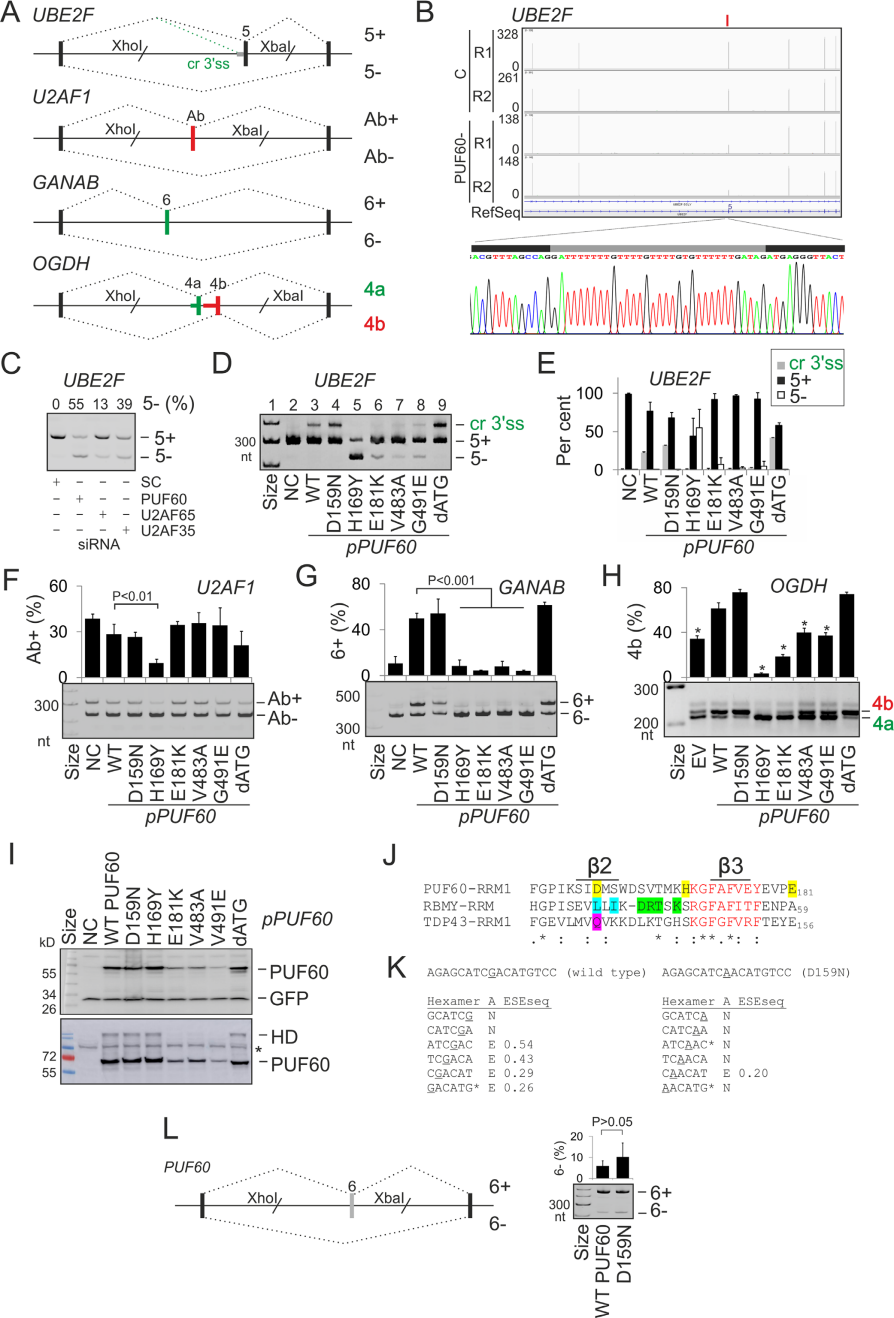
Splicing outcomes of PD alleles. (**A**) Schematics of PUF60-dependent splicing reporter constructs. Exons are shown as boxes, introns as horizontal lines and canonical/aberrant RNA products (named to the right) as dotted lines above/below the pre-mRNA, respectively. Cr3′ss, cryptic 3′ss. (**B**) A genome browser view of RNA-Seq tracks of *UBE2F* from control (C) and PUF60- cells. For full legend, see Figure 3. RNA product employing Cr3′ss in cells overexpressing PUF60 is sequenced at the bottom. Grey rectangle shows a 33-nt insertion. (**C**) *UBE2F* exon 5 inclusion in cells lacking PUF60, U2AF65 and U2AF35. Protein depletion is shown in Supplementary Figure 6D. (**D**) Splicing of exogeneous *UBE2F* transcripts in cells overexpressing WT and mutated PUF60 plasmids. NC, no plasmid control. RNA products (*right*) were amplified with vector primers PL3 and PL4 (46). PD alleles (*bottom*) are in Table 1. Construct dATG lacked exons 1–2, translating PUF60 from a downstream start codon in exon 3 (panel I), possibly representing the outcome of PD-associated 5′ss mutations of exon 1 (33,34), which lead to a loss of canonical start codon. (**E**) The relative abundance of mRNA products in panel D. Error bars are SDs of two transfections. **(F–H)** Splicing pattern of exogenous *U2AF1* (**F**), *GANAB* (**G**) and *OGDH* (**H**) transcripts in cells overexpressing PUF60 constructs shown at the bottom**. I**, Immunoblots of HEK293 cultures transiently transfected with WT and mutated PUF60. Shown are two independent transfections, one with 30 (*upper panel*) and the other with 60 (*lower panel*) μg of protein lysates in each lane. Membranes were blotted with anti-*myc* and anti-GFP antibodies. HD, homodimers; asterisk, a non-specific band. **J**, Alignments of PUF60-RRM1 (Q9UHX1; aa 129–207), RBMY-RRM (Q15415; aa 8–85) and TDP43-RRM1 (Q13148; aa 104–200) around residues mutated in PD (shown in yellow). RNP1 is in red; RBMY residues contacting an RNA loop and stem (90) are in blue and green, respectively, and a DNA-interacting residue TDP43 Q134 (89) is in magenta. Alignment was with full-length RRMs using Clustal Omega (v.1.2.4). (**K**) Hexamer profile across point mutation c.475G>A (underlined) leading to substitution D159N. ‘A’, assignment of splicing neutral (N) and enhancing (E) motifs; ESESeq scores were determined previously for all hexamers (129). Asterisks denote splicing regulatory elements reported by Goren *et al.* (130). (**L**) *PUF60* reporter. Cloning primers are in Supplementary Table S1. Mutation D159N is encoded by exon 6, its inclusion levels are to the right. Error bars are SDs of two transfections.

Apart from truncating mutations, PD has been associated with missense mutations in PUF60 RRMs/UHM (32–34,36). To examine their impact on splicing of PUF60-dependent exons, we prepared plasmid constructs individually expressing most missense PD alleles reported to date (Table 1). The constructs were co-transfected with four splicing reporters into HEK293 cells. Each reporter gave a distinct splicing outcome in cells overexpressing the WT PUF60, namely, cryptic 3′ss activation of *UBE2F* exon 5, skipping of *U2AF1* exon Ab, increased inclusion of *GANAB* exon 6 and alteration of mutually exclusive splicing of *OGDH* exons 4a and 4b (Figure 7A–H, Supplementary Figure S6 and Supplementary Table S7).

In *UBE2F*, RRM1/UHM mutations failed to activate the cryptic 3′ss (Figure 7D, cf. lanes 2–3 and 6–8), except for substitution D159N, which behaved as the WT (lane 4), and H169Y, which induced exon 5 skipping (lane 5). Both D159N and H169Y proteins were expressed at least as the WT, but each tested UHM mutation and E181K showed a reduced expression on immunoblots (Figure 7I). Expression levels of UHM mutants did not appear to mirror exon 5 skipping levels (cf. lanes 5–8 in Figure 7D and I). In a solution structure of PUF60 RRM1/RRM2 (88), H169 is in the exposed loop between β2 and β3 sheets in the vicinity of the ribonucleoprotein motif RNP1 (KGFAFVEY) in β3 (Figure 7J and Supplementary Figure S11), suggesting that H169Y might impair binding to the guanine-ridden PPT between the two competing 3′ss (Figure 7B; Supplementary Table S7), thus preventing selection of both 3′ss. Although D159 is in a less exposed β2 (Supplementary Figure S11), it is located at the same alignment position as RNA-interacting residues of other RBPs with solved RRM structures, including L38 of RBMY and Q134 of TDP43 (Figure 7J) (89,90). Thus, although D159N was not predicted as pathogenic by Polyphen2 (Supplementary Table S8), was splicing-proficient for each tested reporter (Figure 7D–H), had normal steady-state protein levels (Figure 7I), and, unlike all other PD substitutions, is present in lower organisms (insects and worms), it could still alter RNA binding. Interestingly, the protein sequence optimality score Γ (91) for D159 was the lowest of any residue in two solved PUF60 RRM1-RRM2 structures (Supplementary Figure S12), suggesting that this position could be prone to stabilizing mutations. Importantly, D159N activated the *UBE2F* cryptic 3′ss more than the WT as did a construct lacking exons 1 and 2 (dATG; Figure 7D and E). Because mutation G>A leading to D159N results in a loss of predicted splicing enhancer elements (Figure 7K), we also examined *PUF60* exon 6 inclusion in WT and mutated minigenes, but found no marked increase in exon 6 skipping (Figure 7L).

**Figure 8.**
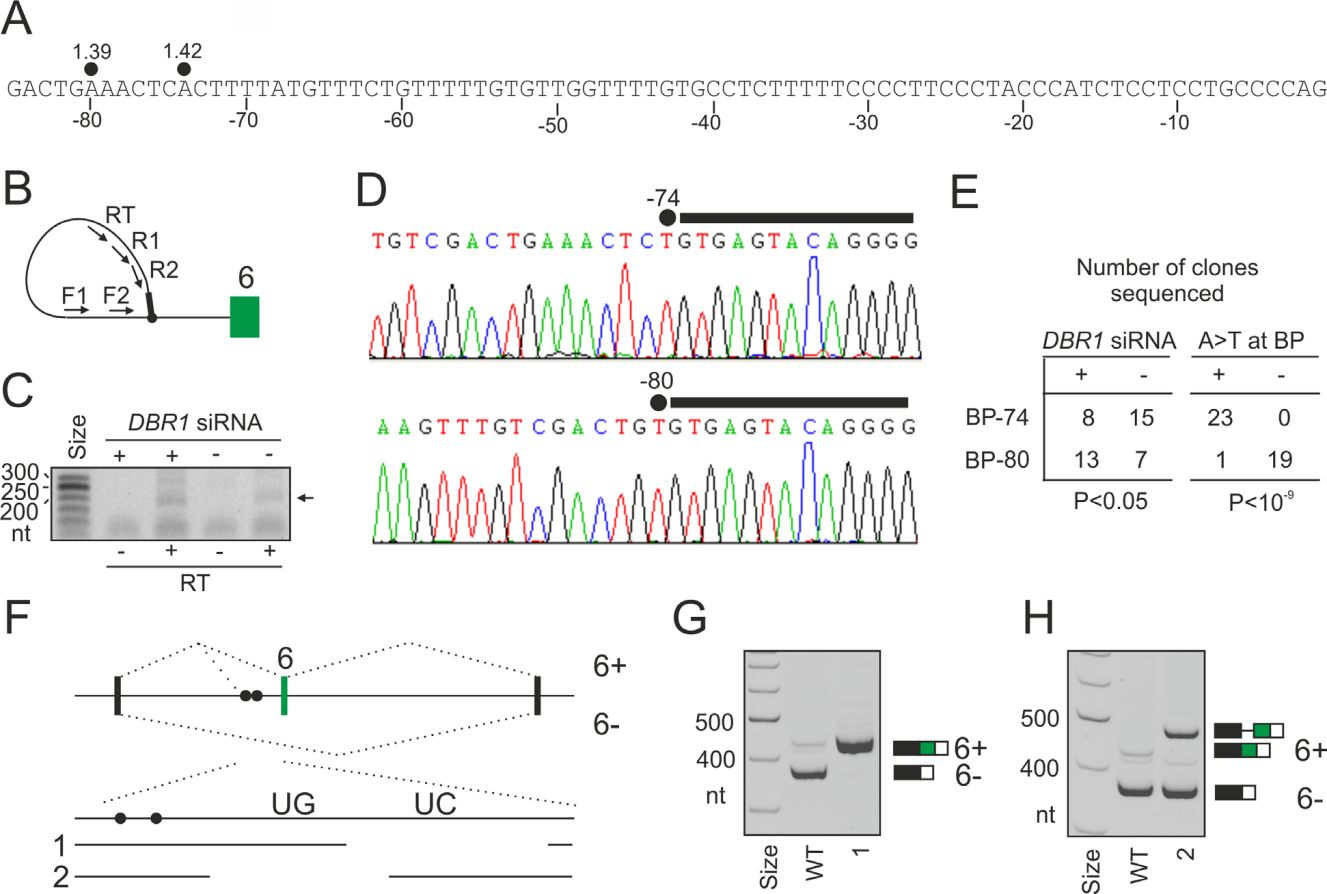
Functional and structural PPT partitioning. (**A**) Nucleotide sequence upstream of *GANAB* exon 6. Predicted BP adenines are denoted by closed circles; numbers indicate their SVM scores (49). **B**, BP mapping primers (Supplementary Table S1). The 5′ end of intron is denoted by a black rectangle. **C**, PCR products amplified from DBR1-depleted (+) and control (–) cultures. Samples were reverse-transcribed in the presence (RT+) or absence (RT–) of reverse transcriptase. Products shown in panel D are denoted by an arrow. (**D**) Representative sequence chromatograms showing A>T mismatches at the lariat junction, which occurs when RT traverses the noncanonical 2′ to 5′ linkage between the 5′ss nucleotide and BP (66,92). (**E**) Distribution of BPs mapped to positions –74 and –80 in DBR1-depleted cells and controls (*left panel*) and with/without A>T substitutions at the lariat junction (*right panel*). *P*-values were derived from Fisher's exact tests. (**F**) Deletions of the UC- and UG-rich segments in the PPT of the WT *GANAB* reporter construct (deletion 1 and 2). Closed circles show two BPs mapped in panels B–D. (**G**, **H**) Splicing pattern of the two deletion constructs after transient transfection into HEK293 cells. RNA products are to the right. Cryptic 3′ss activated by deletion 2 is 91 nt upstream of the natural 3′ss of exon 6 and is schematically shown in panel F.

H169Y also impaired splicing of *U2AF1* exon Ab (Figure 7F), *GANAB* exon 6 (Figure 7G) and *OGDH* exon 4b (Figure 7H). *GANAB* exon 6 was consistently activated in cells lacking U2AF35 or U2AF65 and repressed in cells lacking PUF60 (4,16) while overexpression of WT PUF60, but not PUF60 mutants (except for D159N), stimulated its inclusion (Figure 7G). In full agreement with the SVM BP prediction (49), this exon employed two distant BPs at 74 and 80 nt upstream of the 3′ss (BP-74 and BP-80, Figure 8A–E). Interestingly, BP-80 was preferred in DBR1-depleted cells while BP-74 was more frequent in untreated cells. Unlike BP-80, virtually all BP-74 clones showed substitutions of BP adenines to uridines, which are diagnostic of BPs (66,92). The two distant BPs were just upstream of a 27-nt UG-rich and a 40-nt UC-rich PPT segments (Figure 8A and F and Supplementary Table S7). Deletion of the UC-rich region eliminated *GANAB* exon 6 skipping and produced exclusively canonical transcripts (Figure 8G). By contrast, deletion of the UG-rich region eliminated transcripts spliced to natural 3′ss of exon 6 and activated a cryptic 3′ss just upstream of the BP-80 (Figure 8H). This shows that the UG-rich segment inhibits the cryptic 3′ss and is also critical for selection of BP-74 and BP-80, assuming the cryptic 3′ss requires another BP(s) further upstream. We conclude that the PUF60-activated 3′ss of *GANAB* exon 6 employs two distant BPs, experimentally supporting the shift towards more distant BPs predicted for PUF60-activated exons (Figure 2D). Second, partitioning of the extended PPT into UG- and UC-segments might explain the observed PUF60 activation and U2AF repression through their respective optimal binding preferences.

To test if BP selection is also altered in cells overexpressing PUF60, we set out to map BP(s) of competing 3′ss in the *UBE2F* system (Figure 7A and D). The natural 3′ss of *UBE2F* exon 5 used a BP 36 nt upstream, which is located in the cryptic 3′ss consensus, although it appeared to employ also a BP further 40 nt upstream (BP-76; Supplementary Figure S13A–D). In contrast, cells expressing a PUF60 variant that most strongly activated the cryptic 3′ss employed more BP-76. This indicates that normal PUF60 levels are important for selection of both the 3′ss and the BPs.

In the human PUF60 RRM1/RRM2 crystal structure model (13), E181 (corresponds to E164; Figure 5C in (13)) was implicated in interdomain RRM1-RRM2 contacts. In this model, the PUF60 dimer bound single-stranded DNA through RRM1, with the DNA-binding surface of RRM2 buried in a hydrophobic pocket between the two RRMs, including a tyrosine counterpart of E181. Thus, the reduced expression of PUF60 E181K and functional defects in some but not all tested pre-mRNAs (Figure 7D–I) could be explained by a requirement of RRM2 for PUF60 folding.

**Figure 9.**
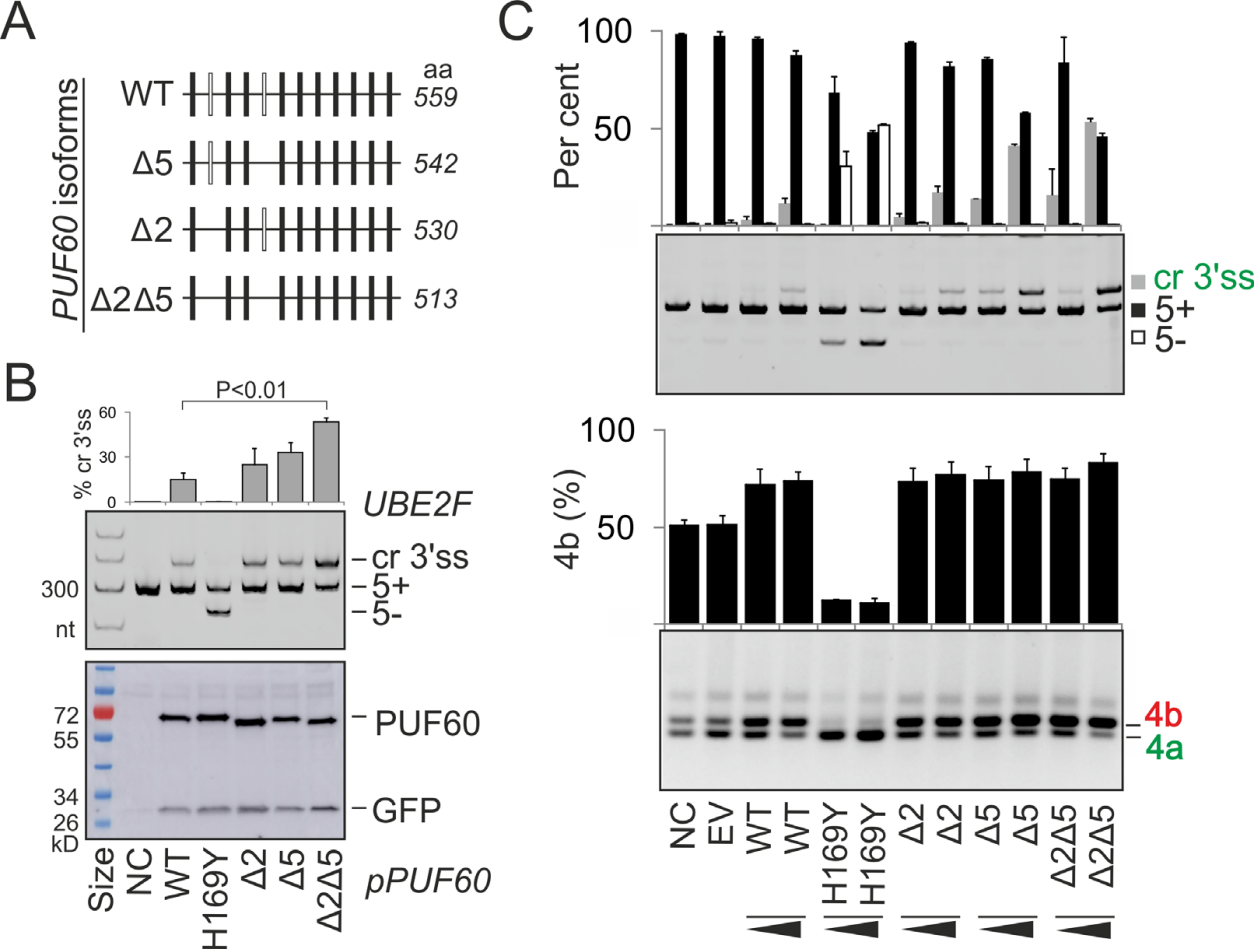
Alternative splicing of *PUF60* affects 3′ss choice. (**A**) Exon structure of tested *PUF60* mRNA isoforms. (**B**) *UBE2E* splicing pattern in HEK293 cells individually expressing PUF60 isoforms (*upper panel*). Their expression was assayed by the anti-*myc* antibodies (*lower panel*). NC, no plasmid control, H169Y, a negative control for cr3′ss. Error bars are SDs of two transfections. *P*-value was derived by an unpaired *t*-test. (**C**) Exon usage of *UBE2F* and *OGDH* reporters (Figure 7A) cotransfected with plasmids expressing PUF60 isoforms. Error bars, SDs of two transfections.

Finally, we examined function of alternatively spliced PUF60 variants that lack exon 2- and/or exon 5-encoded peptides (Δ2, Δ5 and Δ2Δ5 in Figure 9A). These natural PUF60 isoforms were previously identified by RT-PCR and immunoblotting in cell lines and exhibited comparable cancer-promoting properties (19). We observed an increase in cryptic 3′ss utilization of *UBE2F* exon 5 in cells overexpressing the Δ2Δ5 isoform compared to the WT, despite similar expression levels in transfected cultures (Figure 9B). Both exons appeared to contribute to the increased use of cryptic 3′ss, consistent with a similar effect of a PUF60 construct lacking exons 1 and 2 (clone dATG, Figures 7B and 9B). The increase was dose-dependent for *UBE2F*, but was not apparent for the *OGDH* transcript (Figure 9C). The peptide encoded by *PUF60* exon 5 is near RRM1 and contains serines S112 and S116 that are phosphorylated (http://www.phosphosite.org).

Collectively, our results revealed an altered 3′ss and BP selection in cells expressing exogenous PUF60 (Figure 7D,E and Supplementary Figure S13), partitioning of the extended PPT of a PUF60-activated and U2AF-repressed exon into functionally and structurally distinct segments (Figure 8) and functional differences of alternatively spliced *PUF60* isoforms (Figure 9). They also showed that distinct PD-causing substitutions in a single RRM could favour different 3′ss (Figure 7). Whereas the PUF60 UHM and E181K mutations compromised PUF60 expression and selection of the cryptic 3′ss normally promoted by the excess of the WT PUF60, they were insufficient to abrogate selection of the canonical 3′ss. In contrast, H169Y inhibited the use of both 3′ss (Figure 7D and E) and caused the most severe splicing defects of all tested PD alleles (Figure 7D–H). This finding uncovers a novel and surprising complexity of germline RRM alleles in splicing of a single intron.

## DISCUSSION

### 3′ splice sites of PUF60-regulated exons

We have identified a large number of PUF60-activated exons, dramatically expanding a few examples described earlier (15,32). They are preceded by U-rich regions that are most enriched in a typical location of human BPs (Figure 2A and Supplementary Figure S3). They have longer AGEZs (Figure 2F) and their average BPs are shifted further away from 3′ss as compared to control exons (Figure 2D and Figure 8). This 3′ss organization provides a more single-stranded RNA space for ligand interactions, as estimated by the PU values, and also more opportunities for exon usage regulation. Changes in their inclusion levels induced by the PUF60 knockdown significantly correlated with both the extent of singlestrandedness and the AGEZ length (Figure 2F and H), with no difference between the two correlation coefficients (*r* = −0.22 and *r* = −0.23, *P* > 0.05). The extended U-rich segments whose shuffled versions partly but not fully recapitulated the increase in PU values (Supplementary Figure S3) are likely to bind PUF60, in agreement with a requirement for at least 14 nt-long PPTs in mobility shift assays (10). The long PPTs are not functionally homogenous, as suggested by distinct background base frequencies in two MEME submotifs with alternating U frequencies (Figure 2B). The background of the 3′ motif was cytosine-rich whereas the background of the 5′ motif was guanine-rich. The former motif is similar to those bound by U2AF65 or PTB, which also exhibited alternate U patterns (4,93). The 5′ motif is likely to reflect a high-affinity of PUF60 to UG-rich motifs (Supplementary Table S5), which is supported by independent studies. First, PUF60 binding to a PPT was outcompeted by cold poly(U) less efficiently than by poly(GU) at the same concentration (cf. lanes 7, 8 versus 24, 25 in Figure 7 in (10)). Second, recombinant human PUF60 bound a TGTGT pentamer with an affinity exceeding that observed for (T)_5_ (88), also suggesting that RNA and DNA may have the same binding preferences. It is worth noting that PUF60 and U2AF had the opposite effect on guanine accessibility near the BP region (Figure 5C and D in (15)). The observed bipartite pattern (Figure 2B) suggests that PUF60 binds the pre-mRNA upstream of U2AF65, reflecting binding affinities of the two proteins rather than two PUF60 RRMs. This scenario is in an agreement with the observed functional and structural partitioning of the PUF60- and U2AF65-dependent PPT (Figure 8F–H), with PUF60 interacting with the SF3B1-ULM via the C-terminal UHM and with the pre-mRNA in a typical BP location via RRM(s). It is also supported by the previously observed PUF60 binding to a BP region in the *APP* gene (94). The linker between the PUF60-RRM2 and -UHM is the longest among UHM-containing proteins and should ensure flexible contacts with both the SF3B1-ULM and U-rich pre-mRNAs during spliceosome assembly.

In zebrafish, and possibly in many ancestral vertebrates prior to the divergence of tetrapods from teleosts, ∼10% of all introns contain (GT)_n_ tracts upstream of 3′ss that base-pair with (AC)_n_ repeats present downstream of 5′ss, spatially approximating splice sites and obviating the need for U2AF (95). It may be interesting to test if the zebrafish PUF60, which have shorter N-termini than human PUF60 and only the downstream ATG start codon (used by the dATG construct, Figure 7), can supplant the role of structure in the processing of this group of introns.

### Interactions of PUF60 and U2AF with the pre-mRNA

Two U frequency peaks at intron positions –5 and –11 (Figure 2A, *top*) have been thought to reflect U2AF65 binding, which was detected at almost 90% of annotated 3′ss (4). The two peaks in PU values in PUF60-activated 3′ss are at position –8 and –12 and appear to be closer to each other as compared to control 3′ss (Figure 2E), raising a speculation that PUF60 contacts with the pre-mRNA changes U2AF65 binding. Interestingly, RNA protection pattern was altered when PUF60 and U2AF were added to RNase T1 footprinting reactions together (15).

Figure 2 and our previous work (16) showed that U2AF-repressed and PUF60-activated exons had longer PPTs, AGEZs and more distant BPs. The longer AGEZ of PUF60-activated exons was not significantly different from that calculated for U2AF(35)-repressed exons (means 55.5 versus 60.3, *P* = 0.2). This 3′ss organization may provide more space for non-competitive or non-cooperative RNA binding by U2AF65/PUF60 and other stimulatory or inhibitory proteins such as PTB than shorter PPTs, potentially explaining the opposite splicing outcomes. *GANAB* exon 6 probably serves as the most illustrative example (Figure 7G and 8F–H) (4,16), but the binding sites of the two proteins should be confirmed, with U2AF65 binding reported mainly to the UC-region of this PPT by one group (6) and further down to exon 6 by another (4). In contrast, shorter PPTs could favour cooperative interactions of the two proteins and concordant exon inclusion. Synergistic effects of U2AF65 and PUF60 were previously observed for RNAs with shorter PPTs, such as *HBB*. This substrate became PUF60-dependent only when T>G mutations were introduced to the PPT close to the BP at position –32 (15).

PUF60 alone was unable to restore splicing activity of nuclear extracts depleted of poly(U)-binding factors in the absence of U2AF65 in some but not all RNAs and U2AF was not strictly required for splicing *in vitro* when PUF60 was present (10,15). Substrate-specific dependencies on each factor are consistent with a wide range of exon usage alterations for PUF60 and U2AF65 knockdowns (4,16) (Supplementary Table S2), likely reflecting a variety of PPTs and distances between BP and 3′ss.

Unlike PUF60-activated exons, PUF60-repressed exons had weaker PPTs with less Us and more predicted base-paired contacts (Figure 2A, E and I). A putative guanine-rich binding motif suggested by MEME (Figure 2J) could provide a clue to this functional outcome. A highly similar motif (AGGGG) is bound by SRSF11 (https://www.encodeproject.org/experiments/ENCSR073DSH/) (96), which interacts with both PUF60 and RBM39 (10,15,21). SRSF11 also crosslinked upstream of a BP in a PPT-dependent manner (97) and, unlike other SR proteins, interacts with U2AF via U2AF65 rather than U2AF35 (98). G-triples are known intronic splicing enhancers (37,99) that are abundant in mammalian introns but rare in fish (100). Future studies will be needed to test if SRSF11 or other (G)_3_-binding factors can explain the observed higher 3′ss usage in PUF60- cells than in untreated cells.

Overall, human PUF60 may have evolved to promote splicing of introns that acquired longer U-rich tracts that displace BPs to a suboptimal location further upstream (Figure 2). Together with U2AF, PUF60 contributes to the maintenance of exon duplications through their PPT/BP regions (Supplementary Figure S6) (59). Finally, PUF60-regulated exons described here will be useful for establishing causality of new mutations detected in PD syndromes, dissecting the role of other U2AF-related proteins in relation to 3′ss organization and understanding structural requirements in the BP/PPT region for competitive or cooperative interactions with splicing factors.

### Distinct roles of U-binding factors in SINE exonization

The G(U)_*n*_ motifs that bind PUF60 were found also in PPTs of PUF60-regulated SINE exons (Supplementary Figures S8 and S9). Unlike hnRNP C, depletion of TDP43, TIA proteins and HuR did not show increased inclusion of *Alu* exons (6,101), but it remains to be seen to what extent PUF60 contributes to their maintenance in primate transcriptomes. SINE interactions with hnRNP C seem to be crucial to repress *Alu* cryptic 3′ss (6) whereas other U-binding factors might contribute to a smaller subsets of *Alu* exons, such as PUF60 to those with more diverged/longer PPTs with more distant BPs. This notion is supported by the PPT location of most antisense *Alu* exons very close to their 3′ss (102), the U-enrichment of PUF60-dependent exons further upstream (Figure 2A), the PUF60 preference for GU or GT motifs (10,88) (Supplementary Table S5) and decreased splicing repression of ancient *Alu* elements by hnRNP C (6). Future studies should also establish to what extent SINEs and other repeats contributed to PPT partitioning into splicing-repressive and -activating subdomains during evolution.

Among validated events, we did not find free right *Alu* monomers, which are longer than FLAMs and have an extra insertion. Although they are less abundant than FLAMs in the genome, the insertion might promote formation of pre-mRNA structures that reduce or prevent PUF60 binding. Preliminary RNA secondary structure predictions with aligned consensus FLAMs revealed a significant cross-exon complementarity involving stable, ≥7-bp helices, which assist ultrarapid DNA and RNA annealing (103) and were present also in exonized SINEs (Supplementary Figure S8E and F), potentially facilitating formation of scaffolds that could approximate 3′ and 5′ss immediately after transcription before protein binding. Co-transcriptional formation of such cross-exon hairpins was proposed for SINE-derived cryptic exons that were activated by mutations not involving splice sites (104–106).

### UHM-containing proteins in RNA processing

Our results suggest that function of PUF60 and RBM39 is not limited to 3′ss recognition but extends into all RNA processing steps (Figure 1C), consistent with their widespread pre-mRNA binding (4,6,30) and role in transcription (17,20,21). PUF60 depletion influenced many ATI/APA sites, possibly even more than depletion of U2AF subunits (16), but it is not clear if this is due to TFIIH binding (17), strong preferences of stable U2AF heterodimers to the 3′ss consensus (4,5), other UHM–ULM interactions in a growing number of higher order complexes (14), or as yet unknown links between ATI, alternative splicing and APA (107). RNA-Seq studies should not be limited only to splicing of internal exons to avoid unwarranted conclusions about exon usage regulation, particularly for exons located upstream of alternative transcription initiation (ATI) sites and downstream of APA sites. Although current software tools do not accurately describe these events, their integration with ATI/APA resources may provide better alternatives in the future.

Our results also suggest that RBM39 interacts with U1 snRNP via U1-70K (Figures 5 and 6). This interaction requires the N-terminal part with the RS domain, which also supports nuclear localization (Figure 6). Because U1 snRNP prevents premature cleavage and polyadenylation (108), APA alterations in RBM39- cells (Figure 1C) could be potentially explained by reduced RBM39-U1 interactions *in vivo*. Altered APA usage was observed also in U2AF35- cells (16), which associates with RBM39 (Figure 5) (22,27), and in cells depleted of PUF60 (Figure 1C), which contacts U1-70K (15). Nevertheless, it remains to be seen if the RBM39-U1 interaction is stronger than the PUF60-U1 interaction. Because rsd1 links U1 and U2 snRNPs via the Prp5 ATPase (26), the observed APA changes in RBM39- cells could also reflect impaired contacts between U1 and U2 snRNPs, assuming these interactions are conserved in humans.

### U-bound RBP network

Our data support co-regulation of a number of RBPs that have similar binding preferences to PUF60, such as TIA-1/TIAR1 (Figures 3 and 4). In contrast to U2AF, TIA proteins preferentially bind U-rich sequences downstream of 5′ss (109, 110 and references therein). Functional preferences of TIA- and U2AF-related proteins for 5′ss and 3′ss, respectively, could be facilitated by their domain structure. Assuming that the RRM1 of TIA proteins is in fact an UHM (70), the two groups of U-binding proteins, one acting primarily at 5′ss and the other at 3′ss, would have their UHMs at the opposite termini. Their N- and C-terminal UHMs might act as important 5′ and 3′ss anchors of snRNP complexes, respectively, with essential roles of TIA-1-UHM(RRM1) for U1 snRNP binding via interaction with U1-C (111) and PUF60 UHM for the U2 snRNP recruitment (11). C-terminal UHM-containing proteins SPF45 and KIS that lack canonical RRMs were also linked to 3′ss selection (29,112). In contrast, PUF60-regulated hnRNPs that bind U-rich elements but lack UHMs do not appear to show such a conspicuous bias for 3′ or 5′ss, except for hnRNP C and D (6 and ref. therein). On the other hand, we observed long UG repeats downstream of a few PUF60-activated exons (for example, (UG)_22_ in *TMEM175*, which also contains UG repeats in the PPT, Supplementary Table S2) and TIA proteins can bind also upstream of 3′ss (109). In pull down studies, U1-70K was not detected in precipitates with GST-TIA-1, which contained all other U1 components (111). Tandem organization of RRMs was present in most PUF60-regulated U-bound proteins, although this arrangement was not a strict requirement (Supplementary Figure S14).

In contrast to a negative correlation between the 5′ss strength and the presence of downstream pyrimidine-rich stretches in many species, which interact with TIA proteins (85), the intrinsic strength of PUF60-dependent 3′ss was not different from control exons (Figure 2K). This illustrates an inadequacy of 3′ss scoring, historically focused on short sequences rather than the information in longer transcripts and generally favouring U and UC stretches, which are highly variable downstream of BPs also in evolution (113) and can confer splicing repression (6) (Figure 8F and G). Although RRMs have the capacity to bind to almost any dinucleotides using their canonical binding platforms, U residues were the most frequent targets at each registered position in solved RRM-RNA structures (114), suggesting that the RRM evolution in U-binding proteins has been critically important for RNA maturation. Finally, U-rich motifs can also influence the order of intron splicing (115).

### Implications for the phenotypic variability of genetic disease

First, we have shown that distinct disease-causing amino-acid substitutions in the same RRM dramatically influence the choice of competing 3′ss of PUF60-dependent exons (Figure 7). This observation reveals a new layer by which mutation heterogeneity in a splicing factor contributes to the human phenotypic variability. To date, cryptic splice- and/or branch-site activation has been associated with amino-acid substitutions in other domains of human splicing factors, but only in somatic cells (116,117). Mutations in classical RRMs have also been underrepresented among RBD OMIM entries, most likely through lethal effects (118). However, because many RBPs contain two or more RRMs, a single missense mutation may not abolish RNA binding or intramolecular contacts completely and may still be compatible with life. Unlike RRM1, tested UHM substitutions in PD invariably reduced PUF60 expression (Figure 7I), possibly through impaired folding, as shown for a RRM3 substitution in RBM28 in the ANE syndrome (119). Tested residues mutated in PD lack known post-translation modifications and all (except for E181) had a predicted accessible surface area below 30% (http://www.phosphosite.org; http://dbptm.mbc.ntcu.edu).

Second, our findings suggest that disease-predisposing DNA variants located further upstream of a large number of 3′ss may induce splicing abnormalities through altered PUF60 binding. Intronic variants creating or eliminating [(U_n_)G_n_] elements upstream of cryptic 3′ss, including SINE-derived 3′ss, would be prime candidates. They could also modulate repression of *Alu* elements by hnRNP C. Although most disease-causing T>G substitutions upstream of canonical exons produce *de novo* 3′ss by creating AG dinucleotides (120), some do not, yet they activate a cryptic 3′ss nearby (121,122), possibly through altered BP selection. Reduced binding of recombinant PUF60 *in vitro* has been shown for short synthetic RNAs representing a common T>A polymorphism (rs689) in the PPT of *INS* intron 1 (37).

Third, identification of PUF60-dependent genes/exons with OMIM-registered phenotypes as candidate PD modifiers (Supplementary Table S9) should help us understand the clinical variability. For example, at least three genes with PUF60-sensitive exons were associated with mental retardation, including *EDC3, EPS15L1* and *TUSC3*. The MIR-derived, penultimate exon in *TUSC3* contains a canonical stop codon that terminates translation of a longer TUSC3 isoform, which has a distinct C-terminus (Supplementary Figure S8A). Among other U-bound splicing factors regulated by PUF60, Bruno-l4/5 proteins have a similar domain structure as PUF60, with two N-terminal RRMs and a C-terminal RRM3 and a less conserved linker between RRM2 and RRM3, although the RRM3 appears to lack most of typical UHM features. Unlike Bruno-l1-3, Bruno-l4/5 proteins appear to be localized exclusively in the cytoplasm and are expressed mainly in the nervous system, including optical vesicles (123). Heterozygous Bruno-l4 deficiency in the mouse leads to seizures (124), which were described in PD (33). A lack of PUF60 diminished the expression of the Ca^2+^-sensitive OGDH isoform (Supplementary Figure S6), which could render the oxoglutarate dehydrogenase complex dysfunctional, contributing to metabolic and neurological symptoms (Supplementary Table S9). Polycystic kidneys, also a part of the PD syndrome (32,33), were recently associated with a loss of GANAB (subunit α of glucosidase II) (125,126), which is required for maturation of polycystin proteins and their localization to the cell surface (125). Both animal and *S. pombe* mutants were viable (125 and refs. therein). The mRNA isoforms with and without exon 6 are about equally represented in liver and kidney (125), nevertheless the larger GANAB protein was expressed at much lower levels in cell lines (127). *GANAB* exon 6 encodes 22 aa that interrupt a rather unique and disordered B1 subdomain, which is specific to glucosidase IIα and not present in other members of the glycosyl hydrolase 31 family (128). This may influence interactions with the β subunit and the relative abundance of α1β and α2β heterodimers, which have distinct functions (127). Finally, multisystemic PD could be significantly shaped by genes with PUF60-dependent exons preferentially involved in certain functional pathways such as membrane skeleton organization (Supplementary Figure S10) and by genes whose transcription is repressed by PUF60 (17).

## DATA AVAILABILITY

RNA-Seq data are available at ArrayExpress (E-MTAB-6010).

## Supplementary Material

Supplementary Data
