# Supplementary material for: PUF60-activated exons uncover altered 3′ splice-site selection by germline missense mutations in a single RRM
Source: Nucleic Acids Res. 2018 May 18;46(12):6166–87. doi: 10.1093/nar/gky389 (PMC6093180; doi:10.1093/nar/gky389)
Supplement: Supplementary Data [file gky389_supplemental_files.zip › 60-ms-SUPPLEMENT-R.pdf]

Supplemental data

PUF60-activated exons uncover altered 3' splice-site selection by germline missense mutations in a single RRM

Jana Kralovicova[1,2], Ivana Sevcikova[2,] Eva Stejskalova[3], Mina Obuca[3], Michael Hiller[4], David Stanek[3], Igor Vorechovsky[1]

[1]University of Southampton
Faculty of Medicine
Southampton SO16 6YD
United Kingdom

[2]Slovak Academy of Sciences
Centre for Biosciences
840 05 Bratislava
Slovak Republic

[3]Czech Academy of Sciences
Institute of Molecular Genetics
142 20 Prague
Czech Republic

[4]Max Planck Institute of Molecular Cell Biology and Genetics
and Max Planck Institute for the Physics of Complex Systems,
Dresden, Germany

Table S1 Primers

Oligonucleotide	Sequence (5'-3')
**DNA Cloning**[1]	
UBE2F-F	ATTACTCGAGCTGTTCTGAAGGCTGCATCA
UBE2F-R	ATTATCTAGAGTTCCACCAAAGCAAAGACA
PUF60-F	ACCACTCGAGAGGCCTGTCACCAGCTCTAA
PUF60-R	ACCATCTAGAACAAAAGGCTTCCGTGGAG
PUF60-e6mut	ATCAAGAGCATCAACATGTCCTGGGA
GANAB-F	ATAGAATTCGGCTTTCTGTCTCTGGTCGT
GANAB- R	ATATCTAGAACTTGCCTTGTCGCCATCC
PVR-F	ACCAGAATTCTGTCTCTCCCAGGACCAT
PVR-R	ACCATCTAGACACCCTCCTTAGCTCCAACA
OGDH-F	ATTACTCGAGTTTGGTTTCTGGTGGCATGG
OGDH-R	CAGGTTAGCAGCACAGTGAC
**Validation**	
TIAR-E2	CCTTTCCAGAGATGTGACAGAA
TIAR-E4	GGTGTGGTTGCCCAGTTTAC
TIAR-E5	ATCACTTCCATGTGTTTGTTGG
TIAR-E6	TAAAAAGATACAAAACCATAGCCTTT
TIA-1-E5F	TCAAAGTGAATTGGGCAACA
TIA-1-E5R	CTTCCAAATGGTGCAAAAGC
TIA-1-FcrE	TTTCCATGTCTTTGTTGGTGA
TIA-1-RcrE	CCATTTGTTGAAAAAGGAGACA
TIA-1-Re8	GGGTTGCCCAGTTAGTTCTG
HNRNPC-F	GATCCTCGCTCCATGAACTC
HNRNPC-R	CCCGTTGAAAGTCATAGTCCA
HNRNPD-F	AGCCATGTCGAAGGAACAAT
HNRNPD-R	TGTAGCTATTTTGATGACCACCTC
HNRNPK-F	CGGGAGCTTCGATCAAAAT
HNRNPK-R	GCAGGACTCCTTCAGTTCTTC
MATR3-F	CAGGAACCTAATATGCTTCTTGA
MATR3-R	CCATCTGCGTTTTCACTTGT
UBE2F-F	GCATTTTCCTGATCCAAACAA
UBE2F-R	CCCTGTCTCTGTGATGTTGG
PVR-F	TCCTGTGGACAAACCAATCA
PVR-R1	AGTCAAATGGGGGAAACCTC
PVR-R2	GCGCTGGCATGCTCTGTA
OGDH-RT-F	GCACAGTCCCTGGTAGAAGC
OGDH-RT-R	GCAGAGGAAGTGCTGATTCC
**SINE exons**	
DLGAP5-F	TCACTACTGAATGCCACCTTCT
DLGAP5-R	AAATTCAAAATTCTCCTGGTTG
EDC3-F	GAATTCTTCTGTGGGTCCTCTG
EDC3-R	ACACCCAAGCTATCTCCACAAT
ZFAND5-F	CCTCATCGTGCCGGCTT
ZFAND5-R	TGGGCTCATTCTGCCACTAT
TUSC3-F	GGCCTGGTGGTCTTCTTCTT
TUSC3-R	AAATTTACTAGGAAAACCCACAGTTT
ECOL-F1	AAGTGAGCGACACACTCTGC
ECOL-F2	TCTGACCCAGCGGAAGTAAT
ECOL-R	CAATTTGACATACATGGCATCA
**Branch point mapping**	
GANAB-F1	AGCTGTACCCTGGGCATCTC
GANAB-R1	GCTGTCCCACCACCGTCTC
GANAB-F2	TCCCCTTCTCACATCATCA
GANAB-R2	CCTCACAACACCCCTATGC
GANAB-RT	GTGACTGCCTCCTGGTCT
UBE2F-F1	GAACATGGTAGGATGCAAGGA
UBE2F-R1	TCGATCACCTGCCTCACTATT
UBE2F-F2	CATGGAGCCCATTTCTGTTC
UBE2F-R2	CAAATGGAAAATACAACTACG

[1]Mutagenic primers for PD alleles are in Table 1.

Table S2 PUF60/RBM39-regulated exons, control exons and examples of exons concordant and discordant for PUF60 and U2AF

(supplementary excel file)

Table S3 Summary of changes in transcripts coding for UHM- and ULM-containing proteins in HEK293 cells depleted of PUF60 or RBM39

**Cells**	***U2AF1***	***U2AF2***	***RBM39***	***RBM23***	***PUF60***	***RBM17***	***SF1***	***SF3B1***
PUF60-	Upregulation (1.4x), skipping of exon Ab	Downregulation (0.6x)	1.3x; exon 3 skipping[1]	1.1x, skipping of two exons	0.1x	1.2x	0.9x	1.0x
RBM39-	Upregulation (1.7x)	Upregulation (1.5x)	0.4x	1.1x	1.3x	1.3x	Upregulation (2.3x), shortening of 3'UTR[2]	1.2x

[1], the same poison exon was skipped in cells lacking U2AF35 (1). [2]activation of alternative 3'ss of the last exon.

Table S4 MEME motif location upstream of PUF60-activated exons

Intron id	P value[1]	Motif
23	2.38e-8	GGCUUUUUUUUUUUUGUCUUUCUCUCUC
3	2.92e-8	GUUUUUUUUUUUUCUUUUCUUUUCUUUU
56	1.30e-7	CUGCUUUUUGUUUGUUUCCUUUUCUUUC
72	1.54e-7	GCCUCUGCUUUGUGUUUCCCUGUGUGUC
78	1.82e-7	GCCUUUUUUUUUUUUUUUUUUUUUUUUU
8	1.82e-7	GUGUUUGCUUUUUGUUUCCUUUUUUUUU
74	2.15e-7	GCCAUUUUCUUUUCUUUUCCUUUGUUUU
44	4.04e-7	CAUAUUUCUUUCUUUCUCCUUUUUUUUU
79	9.62e-7	CUCUUUUCUCUCCCUCUCCCUCUCUCUC
67	9.62e-7	CAUCUUGAUUUCUCUUUCUCUCUCUCUC
49	1.26e-6	GAUGUUCUUUUUCUUUUUCUUUUCUUUU
47	1.86e-6	CUUUUUUUUUCUUCUGUCCUUUUGUGUG
36	1.86e-6	GCCUUGCUUUUUUUUUUUUUUUUUUUUU
32	2.11e-6	CUCUUUUUUUUUUUUUUUUUUUUUUUAC
13	2.70e-6	GAUUUUUUUGGUUUUCUCUUUCUCUUUG
48	3.05e-6	CUCAUUUUCUUUUUUUGUCUCUUCUGUC
39	3.05e-6	CUGCUUGUUUUGUGUUUGUCUUUUUCUU
84	3.43e-6	CUCCUUUUUGUUUCUGCCCUUCUGUGAU
71	3.43e-6	CCUUUUUUUUUUCUUUUUCUUCCCUCUU
65	3.43e-6	GUUAUUGAUUUUUCUGCUUCUCUCUCUC
5	4.33e-6	GUUGUUGUUGUUUGUUUGUUUUUCUGUA
40	6.75e-6	GCCCCUGCUCUCCCUCUCCCUUUCUCUU
46	7.51e-6	CUCACUUUUAUGUUUCUGUUUUUGUGUU
29	7.51e-6	CCCAUAGGUUUGUGUUUGUUUGUUUGUU
85	1.03e-5	GUUUUUCCUUUUUUCUUCUUUUUUUUUU
92	1.25e-5	GACUUGGCAGUGUUUCCUUUUCUUUUUC
15	1.25e-5	GACAUUUAUUUGUCUUGUUUUUGCUUUU
87	1.52e-5	CCUUUUCUAUUGUCUUUGUCUUGCUUUU
99	1.68e-5	AACUGUUGAUUCUUUCUCCCUAUGUCUC
77	1.68e-5	CUGUUUUUUUUGUAUUUUGUUUUCUUUC
59	1.84e-5	CAUACUGUUUUUUGUUUUUUUUUUUGAG
1	1.84e-5	GUUUUGUUCUUUUCUCCCCUCCUGUUUU
35	2.02e-5	CACUGUGUUUAUUCUUUGCUUUCCUCUC
22	2.21e-5	GCUAUUUUAUUUUGUUUUUUAUUGUUUU
58	2.65e-5	CAUCUUCUAUUUUUUGUCUCUUUUUUUA
61	2.89e-5	ACCUUUGUUAUCUUUGUUUUUGUUUUUC
37	2.89e-5	CUCGUUUUUUUCCUCCUCCUUCCCUCUU
21	2.89e-5	CCUUUUGGAUUUUUCCCCCCUUUGUUAC
88	3.15e-5	AUUUUUUUUUUUUUUGGCAUUAUCUUUU
55	3.74e-5	GUCAUUUGUAUAUAUUUCUUUUUGUCUG
81	4.41e-5	GUUAGUGCACUUUUUCUUCUUCUCUUUA
17	5.18e-5	CCUUUUUUCUUUCAUCUCCCUGUUUCUG
50	6.07e-5	AACAUUUUAUCAUUUUUCUUUCUUUUUU
24	6.07e-5	CCCCCUCCCGUGUCUGGUCCUAUCUCUC
2	6.07e-5	GAUUUCUUUUCUCUUUCCCUCUUCUCUC
16	8.82e-5	AAUUUCUGAUGGUCUCUCUCUCUCUGUC
9	9.48e-5	GUUUUGUUGUUGUGUUGUGUUUUCUCUU
38	1.17e-4	AGCAGGGGUCUGUGUUUCUUUGUCUCAC
28	1.17e-4	CAUGUACUUAUGUGUGCUCUCUUCUUUU
27	1.17e-4	GGUAUGUUUUUGUUUUUCUUCAUUUUAU
76	1.85e-4	CCCGUGCCUUGUUGUGUGUUCUUUUCUG
57	1.85e-4	GUGUUUUGUAUUUGUUAUAUUUUCUGUU
90	1.97e-4	UACUUUGUUGUUUUUUGGAUUAUCUUUU
14	2.37e-4	GACAGAGGUGCUCCCUGCCUUCUGUGUU

The motif is shown in Fig. 2B (*upper panel*). [1]The probability that an equal or better site would be found in a random sequence of the same length. The number of intronic sequences contributing to the motif construction was 54.

Table S5 Motif enrichment analysis of 100 nt-sequences upstream of PUF60-activated 3'ss

Enriched motif[1]	Corrected P-value[2]	RNA-binding protein
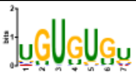	0.004	Bruno-l5 (CELF5)
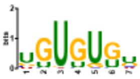	0.005	Bruno-l4 (CELF4)
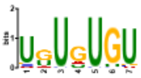	0.02	Bruno-l1 (ARET)
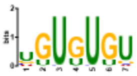	0.03	PAPI

[1]Web logo. [2]Wilcoxon rank-sum test Bonferroni correction, as implemented in AME (total odds scores, background Markov model). The number of multiple tests was equal to 68 RBP motifs identified previously (2).

Table S6 SINE exons containing $[(T)_nG]_n$ repeats in the PPT

(supplementary excel file)

Table S7 Organization of PUF60-dependent 3'ss in *UBE2F*, *U2AF1* and *GANAB*

Exon	3'ss	Sequence[1]	PUF60 WT overexpression	BP position	AGEZ (nt)
UBE2F exon 5	Cryptic	gcatttggtgatggaaccgaggaactctgcaggcctgtttttaaactcctgattctctgta<br>tttgccatatcctaAcagagtgtttgctttttgtttcctttttttttttaatttaaag/GAT	Cr. 3'ss activation	-43	37
UBE2F exon 5	Canonical	cctgtttttaaactcctgattctctgtatttgccatatcctaacagagtgtttgctttttg<br>tttcctttttttttttaAtttaAaggattttttgttttgttttgtgttttttgatag/ATG	Repression	-41, -36	33
U2AF1 exon Ab	Canonical	tgacttgcgggaaaaaaacaaacgtttagccatttgcaaacaaattgtctctttgcaattg<br>tctaatatatgcacagcAgccagtAgAAttccccttttatttttttttccccgcag/ACC	Repression	-30, -31, -33, -40	31
GANAB exon 6	Canonical	gtttctttttccccccatctctggaagtttgtcgactgAaactcActtttatgtttctgtt<br>tttgtgttggttttgtgcctctttttccccttccctacccatctcctcctgccccag/TTT	Activation	-74, -80	91

[1]BPs are in capitals highlighted in grey. Intron-exon junction is denoted by a slash. Cryptic 3'ss AG in *UBE2F* is highlighted in blue. *U2AF1* exon Ab BPs were determined previously (3). Of two *GANAB* exon 5 BPs mapped in this study (Fig. 8), one (position -74) was reported previously using RNA-Seq data (4). *UBE2F* BPs were mapped in Fig. S13.

Table S8 Polyphen2 predictions for tested PUF60 substitutions

**Substitution**	**Polyphen2 score**[1]	**Sensitivity**	**Specificity**
D159N	0.060	0.94	0.84
H169Y	**0.999**	0.68	0.97
E181K	**0.999**	0.14	0.99
V483A	**0.969**	0.77	0.95
G491E	**0.997**	0.41	0.98

[1]Significant Polyphen2 scores (http://genetics.bwh.harvard.edu/pph2/) are in bold.

Table S9 Genes with PUF60-dependent exons previously associated with human genetic disease

Gene symbol	Gene product	OMIM[1] number	Disease[1]
PTS	6-pyruvoyltetrahydropterin synthase	261640	Hyperphenylalaninemia, BH4-deficient, A
AFG3L2	AFG3 like matrix AAA peptidase subunit 2	610246	Spinocerebellar ataxia 28,614487Ataxia, spastic, 5, autosomal recessive
ALG13	ALG13, UDP-N-acetylglucosaminyltransferase subunit	300884	Congenital disorder of glycosylation, type I
CWF19L1	CWF19-like 1, cell cycle control	616127	Spinocerebellar ataxia, autosomal recessive
DRAM2	DNA damage regulated autophagy modulator 2	616502	Cone-rod dystrophy
TBX18	T-box 18	143400	Congenital anomalies of kidney and urinary tract
VPS37A	VPS37A, ESCRT-I subunit	614898	Spastic paraplegia, autosomal recessive
WNK1	WNK lysine deficient protein kinase 1	201300	Neuropathy, hereditary sensory and autonomic, type II
APBB2	amyloid beta precursor protein binding family B member 2	104300	Alzheimer disease, late-onset
APOPT1	apoptogenic 1, mitochondrial	220110	Mitochondrial complex IV deficiency
ASPH	aspartate beta-hydroxylase	601552	Traboulsi syndrome
DGUOK	deoxyguanosine kinase	251880	Mitochondrial DNA depletion syndrome (hepatocerebral type)
DNM2	dynamin 2	160150	Myopathy, centronuclear
EDC3	enhancer of mRNA decapping 3	616460	Mental retardation, autosomal recessive
EPB41L1	erythrocyte membrane protein band 4.1 like 1	614257	Mental retardation, autosomal dominant
EPB41	erythrocyte membrane protein band 4.1	611804	Elliptocytosis-1
FN1	fibronectin 1	601894	Glomerulopathy with fibronectin deposits 2
GAD1	glutamate decarboxylase 1	603513	Cerebral palsy, spastic quadriplegic
GOSR2	Golgi SNAP receptor complex member 2	614018	Epilepsy, progressive myoclonic
GPR98	G-protein coupled receptor 98	605472	Usher syndrome, 2c
LAMP2	lysosomal associated membrane protein 2	300257	Danon disease
NPHP3	nephrocystin 3	208540	Renal-hepatic-pancreatic dysplasia
NF2	neurofibromin 2	101000	Neurofibromatosis, type 2
OGDH	oxoglutarate dehydrogenase	203740	Alpha-ketoglutarate dehydrogenase deficiency
PSAP	prosaposin	249900	Metachromatic leukodystrophy due to SAP-b deficiency
PPM1K	protein phosphatase, Mg2+/Mn2+ dependent 1K	615135	Maple syrup urine disease, mild variant
RBPJ	recombination signal binding protein for immunoglobulin kappa J region	614814	Adams-Oliver syndrome
SLC25A19	solute carrier family 25 member 19	607196	Microcephaly
TMPO	thymopoietin	613740	Cardiomyopathy, dilated
TUSC3	tumor suppressor candidate 3	611093	Mental retardation, autosomal recessive

[1]Online Mendelian Inheritance in Man (http://omim.org)

Figure S1 Examples of altered RNA processing events in PUF60- or RBM39-depleted cells

Genome browser views of RNA-Seq tracts from control (C) and depleted (PUF60- or RBM39-) cells (2 biological replicates each). Downregulated and upregulated exons are denoted by red and green rectangles. **A-B,** exon skipping; **C-D,** up- and down-regulation at the transcript level with no apparent change in exon usage; **E-F,** altered gene termination of splicing factor 1 (*SF1*) **(E)** and poliovirus receptor (*PVR)* **(F).** *PVR* transcripts generate four alternatively spliced isoforms (α, β, γ and δ; schematically shown at the top) that produce transmembrane isoforms α and δ, which bind to poliovirus, and soluble isoforms β and γ, which reduce polivirus entry to the cell (5). **G,** Both PUF60 and RBM39 are required for *PVRδ* but have opposite effects on the relative expression of soluble isoforms *PVRβ* and *PVRγ*.

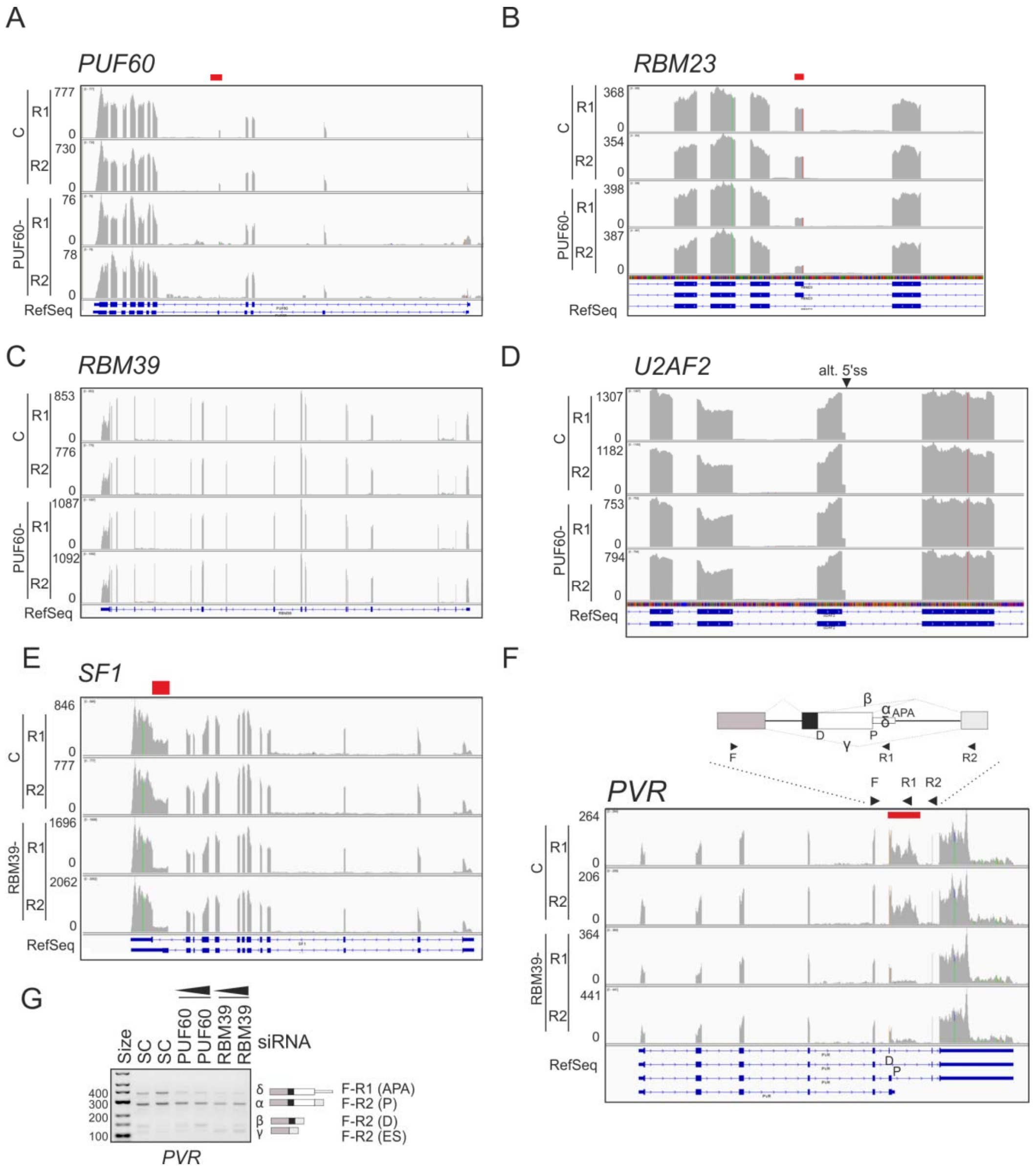

Figure S2 Introns flanking PUF60-activated exons

A-B, Information content of the extended region upstream of 102 PUF60-activated exons **(A)** and downstream of their 5'ss **(B)**. **C,D,** MEME of intronic flanking sequences (+7 to +100 nt) downstream of their 5'ss. Two most significant motifs are shown; E values were computed as described (6).

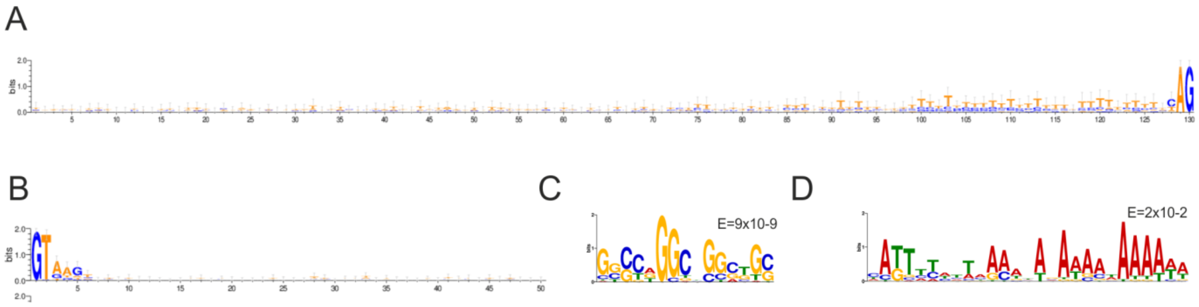

Figure S3 Mean PU values in extended regions upstream and downstream of PUF60-regulated exons and control exons

Exon-intron boundaries are indicated by vertical dotted lines. In the inset at the bottom, positions involved in extended U1 snRNA–5'ss base-pairing interactions are highlighted in grey, allowing for shifted/bulged registers (7). Horizontal rectangles denote significant differences in means between the indicated exon groups (shown to the left). P-values were computed by two-sided Wilcoxon-Mann-Whitney tests. For full details, see the main text and Fig. 2 legend.

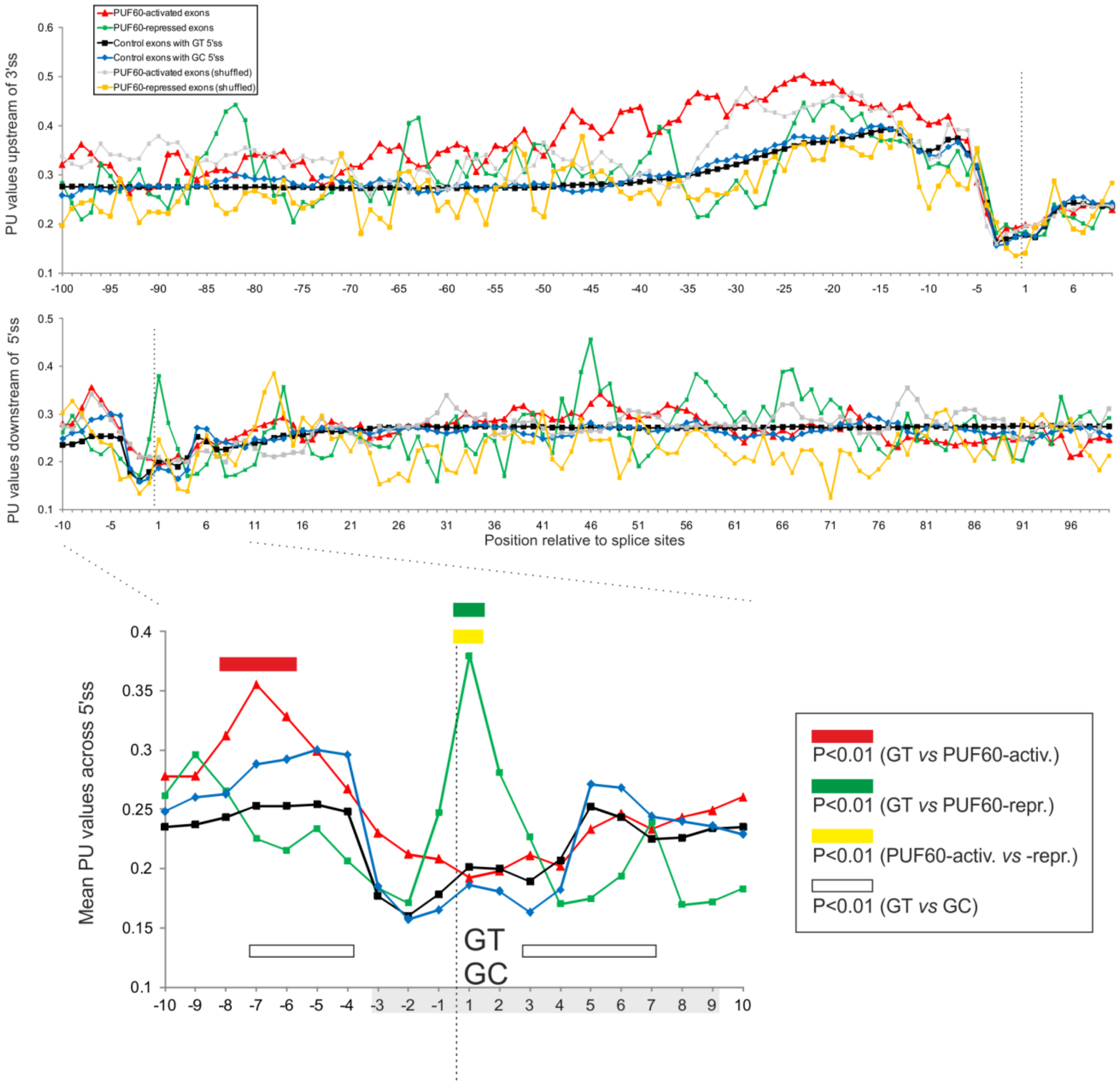

Figure S4 Information content upstream of RBM39-activated 3'ss

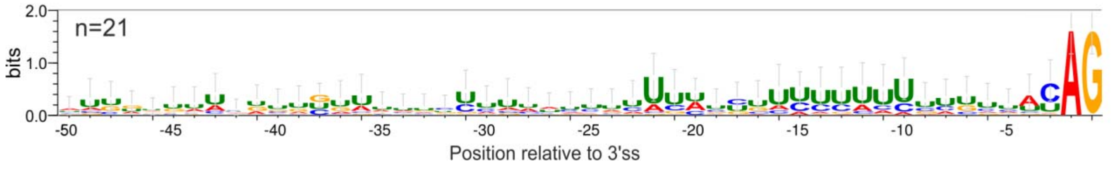

Figure S5 Examples of PUF60-activated and U2AF-repressed exons

RNA-Seq tracks are shown as Sashimi plots of *OS9*, *PICALM*, *PPHLN1* and *PLOD2* transcripts. Oppositely regulated exons are denoted by rectangles at the top. Additional examples are in Table S2.

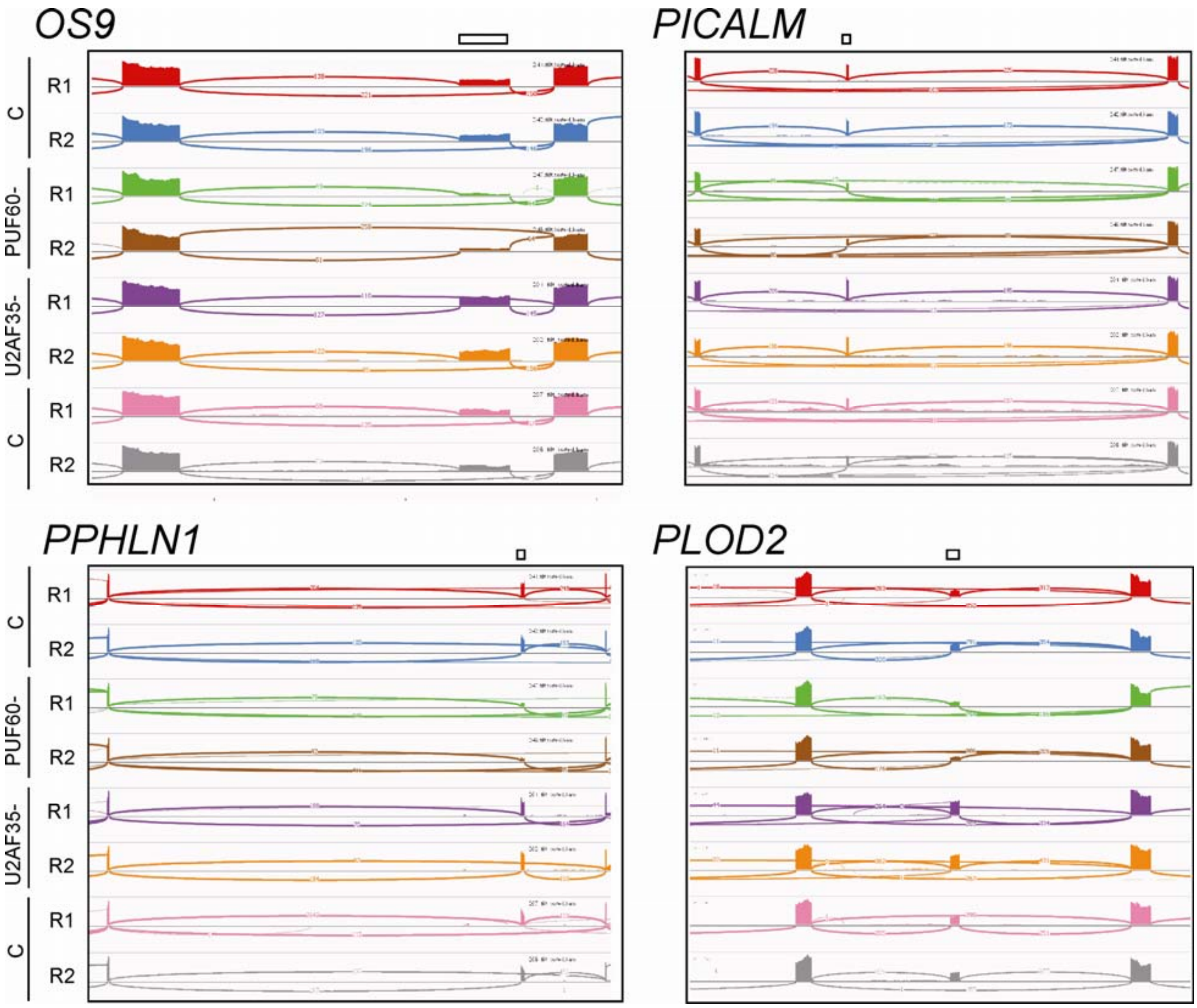

Figure S6 Regulation of mutually exclusive *OGDH* exons 4a and 4b by PUF60 and U2AF
A, Genome browser views of RNA-Seq reads obtained from HEK293 cells lacking PUF60 or U2AF35 and control cells (C). For a full legend, see Fig. 3. **B,** BP/PPT region of duplicated exons 4a and 4b (*upper panel*) and their pair-wise alignment (*lower panel*). Predicted BPs with positive SVM scores are highlighted in grey. Intronic sequences are in lower case, exons are in upper case. **C,** Validation of exon inclusion in endogenous (*upper panel;* primers OGDH-RT-F and OGDH-RT-R, Table S1) and exogenous (*lower panel,* primer PL4 with a transcript specific primer) mature transcripts in HEK293 cells lacking PUF60 or U2AF subunits and in cells transfected with scrambled controls (SC). Minigene construct is shown in Fig. 7A. Asterisk indicates endogenous RNA products containing a cryptic exon (termed 'insert exon' in ref. (8)), which was observed upon U2AF35 knockdown. **D,** Western blot analysis of depleted cells. SDS-PAGE (*upper panel)* was blotted onto a nitrocellulose membrane, cut in the middle and incubated with the indicated antibodies (*lower panel*).

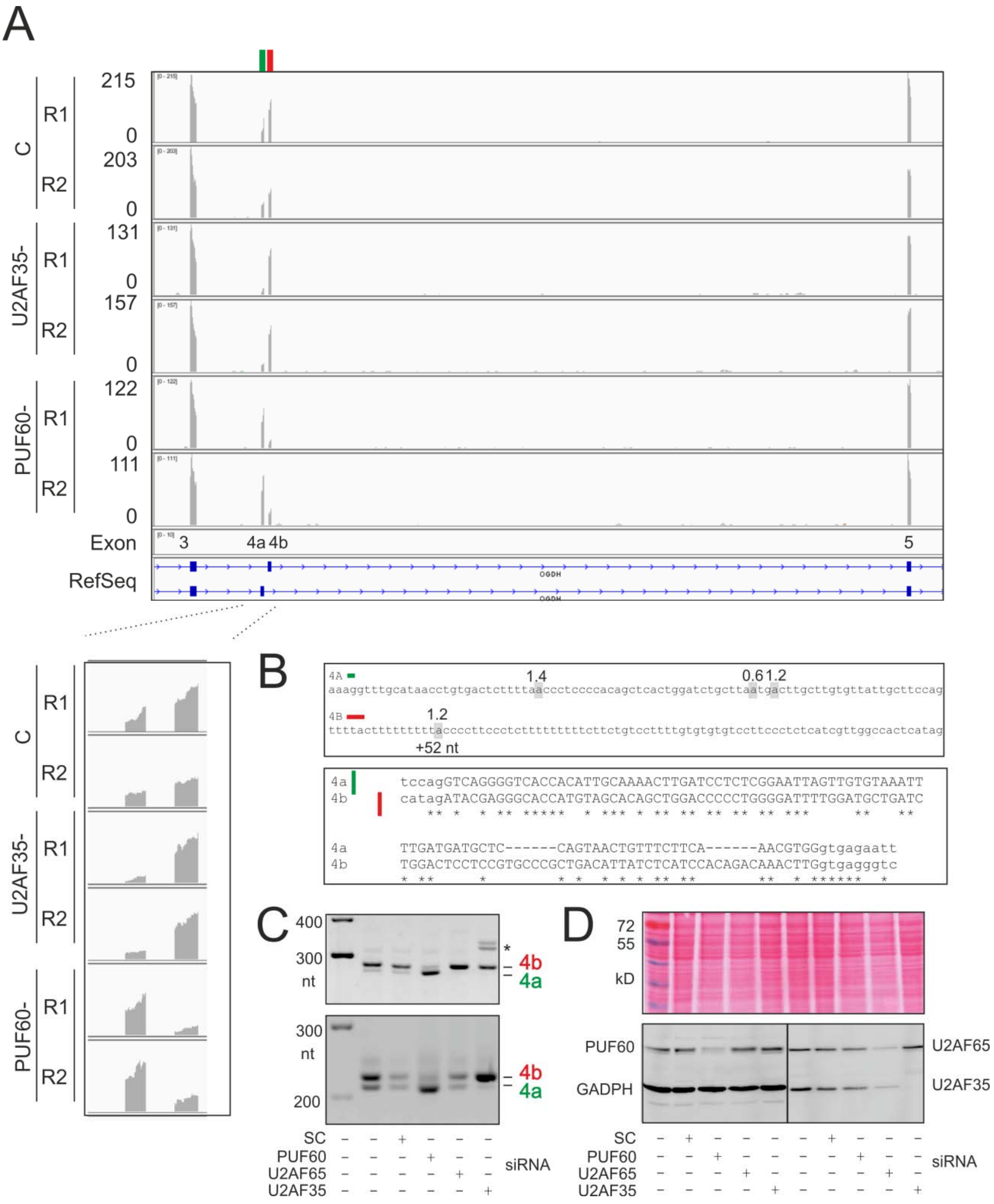

Figure S7 Alternative splicing of *HNRNPM* is regulated by PUF60

For a legend, see Fig. 3.

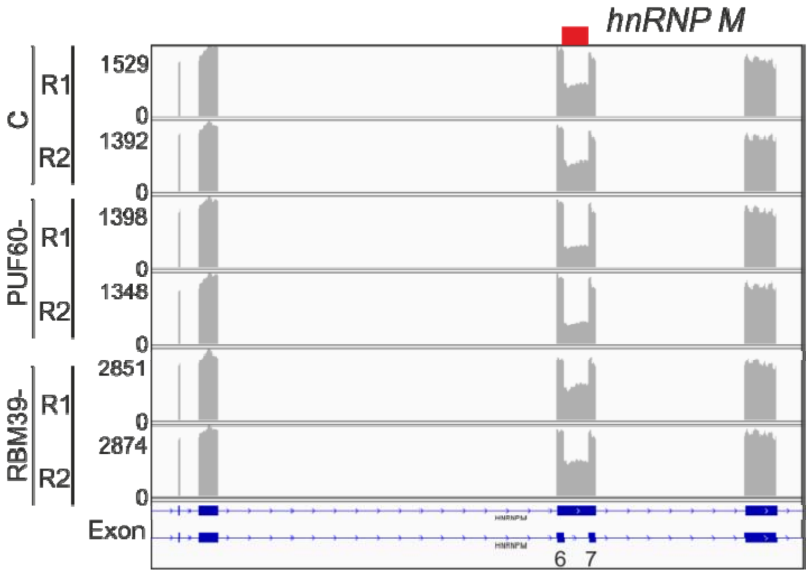

Figure S8 PUF60-activated SINE exons

A, Alignments of PUF60-activated SINE exons in *DLGAP5*, *EDC3*, *ZFAND5* and *TUSC3* with SINE consensus sequences. Exons are in upper case and highlighted in grey, introns are in lower case; v and i denote transversions and transitions, respectively. The first three transposed elements are in antisense orientation, MIRb is in the sense orientation. Canonical stop codon in *TUSC3* is highlighted in yellow. **B,** Genome browser views of RNA-Seq tracks with PUF60-activated SINE exons (red rectangles at the top) and their RT-PCR validation in independent PUF60 depletion experiments. Primers (Table S1) are denoted by arrowheads. ES/EI, exon skipping/exon inclusion. **C,** Frequency distribution of *Alu* families in human *Alu* exons with $[(T)_nG]_n$ repeats in their PPTs**. D,** Example of a PUF60-dependent *Alu* exon with $[(T)_nG]_n$ PPTs not detected by stringent DEXSeq analyses. **E, F,** Most stable RNA secondary structures predicted for PUF60-dependent SINE exons. 5' and 3'ss are denoted by red and blue arrows, respectively, and putative rapidly annealing helices by black rectangles. **E,** FLAM, subfamily A (*upper panel*) and its *DLGAP5* copy (*lower panel*). **F,** FLAM, subfamily C (*upper panel*) and its *EDC3* copy (*lower panel*).

A

```
DLGAP5               ttgtttgtttgtttttgagGTGGGATCTTGCTATGTTGCTCGGGCTGGTC
                       v   v   v    v   ii   i   i          i i
FLAM_A#SINE/A        TTTTTTTTTTTTTTTTTAGAGACGGGGTCTCGCTATGTTGCCCAGGCTGGTC

DLGAP5               TTGAAGTCCTGGGTTCAAGTGATCCTACCACCTCAGCCTCCCGAGTAGCT
                      i   v       i     i      v  i
FLAM_A#SINE/A        TCGAACTCCTGGGCTCAAGCGATCCTCCCGCCTCAGCCTCCCGAGTAGCT

DLGAP5               GGGACTACAGgtgtgcaccaccgcacccgg
                                i i  i       i
FLAM_A#SINE/A        GGGACTACAGGCGCGCGCCACCGCGCCCGG


EDC3                 tttttgtttttttttttgagacagGGTCTTGCTGTGTTGCCCAGCCTGGT
                          v          v     i      i   i          v
FLAM_C#SINE/A        TTTTTTTTTTTTTTTTTAGAGACGGGGTCTCGCTATGTTGCCCAGGCTGGT

EDC3                 CTTGAATTCCTGGACTCAAGTGATGCTCCTGCCTTGGCTTCCCAAACTCC
                       i   i      i      i   v    i    i   i       v v
FLAM_C#SINE/A        CTCGAACTCCTGGGCTCAAGCGATCCTCCCGCCTCGGCCTCCCAAAGTGC

EDC3                 TGGAATTACAACTgtgagccactgcacccagcc
                        i      ivi         i  i   i
FLAM_C#SINE/A        TGGGATTACAGGCGTGAGCCACCGCGCCCGGCC


ZFAND5               CTGAAAAGGAGCCTTTAAAAGTC----AATTCTACTC---CATTTGGCAA
                        i  i   -- i   i ii  ---- i i v  i ---     vi  i
MIR3#SINE/MIR        CTGGAAGGGA--CCTTAGAGATCATCTAGTCCAACCCCCTCATTTTACAG

ZFAND5               A-GAGGGAACTGAAATCTGGAAAG--TAAG-GACTTGCCCAAGGTCAC--
                      -    i      iii ii  i  --v   -                 --
MIR3#SINE/MIR        ATGAGGAAACTGAGGCCCAGAGAGGTGAAGTGACTTGCCCAAGGTCACAC

ZFAND5               --TGACTTAGTGGCAGACTAGAGACTTGAACTCAGGTCTCC--ATTCCCAG
                     --i? v           viv i    v    i         -- i
MIR3#SINE/MIR        AGCKAGTTAGTGGCAGAGCTGGGACTAGAACCCAGGTCTCCTGACTCCCAG


TUSC3                cattgtggtgtattggaaagTGATCTGGACTTTG-AGTGAGA-AGATGTG
                       vi      i v       v -ivi  i     -   v  i-   iv
MIRb#SINE/MIR        CAGCGTGGTGCAGTGGAAAGAG-CACGGGCTTTGGAGTCAGGCAGACCTG

TUSC3                A-TTTGGACCATGGC------ACTTAAAAACTCTATAACCTCAGgcaagt
                     i-  i i i v    ------     vv i  v i i    ii
MIRb#SINE/MIR        GGTTCGAATCCTGGCTCTGCCACTTACTAGCTGTGTGACCTTGGGCAAGT

TUSC3                cttttaatcttctctgagcctcagttttcctcatttttcaaatatagaga
                      vi    i  -                -      i v v    ivi  v
MIRb#SINE/MIR        CACTTAACCT-CTCTGAGCCTCAGTTT-CCTCATCTGTAAAATGGGGATA

TUSC3                gtataacatttatctcataagacaagttgtagtaa
                     --    i ii  i   ii i ----     v  i
MIRb#SINE/MIR        --ATAATACCTACCTCGCAGG----GTTGTTGTGA
```

B

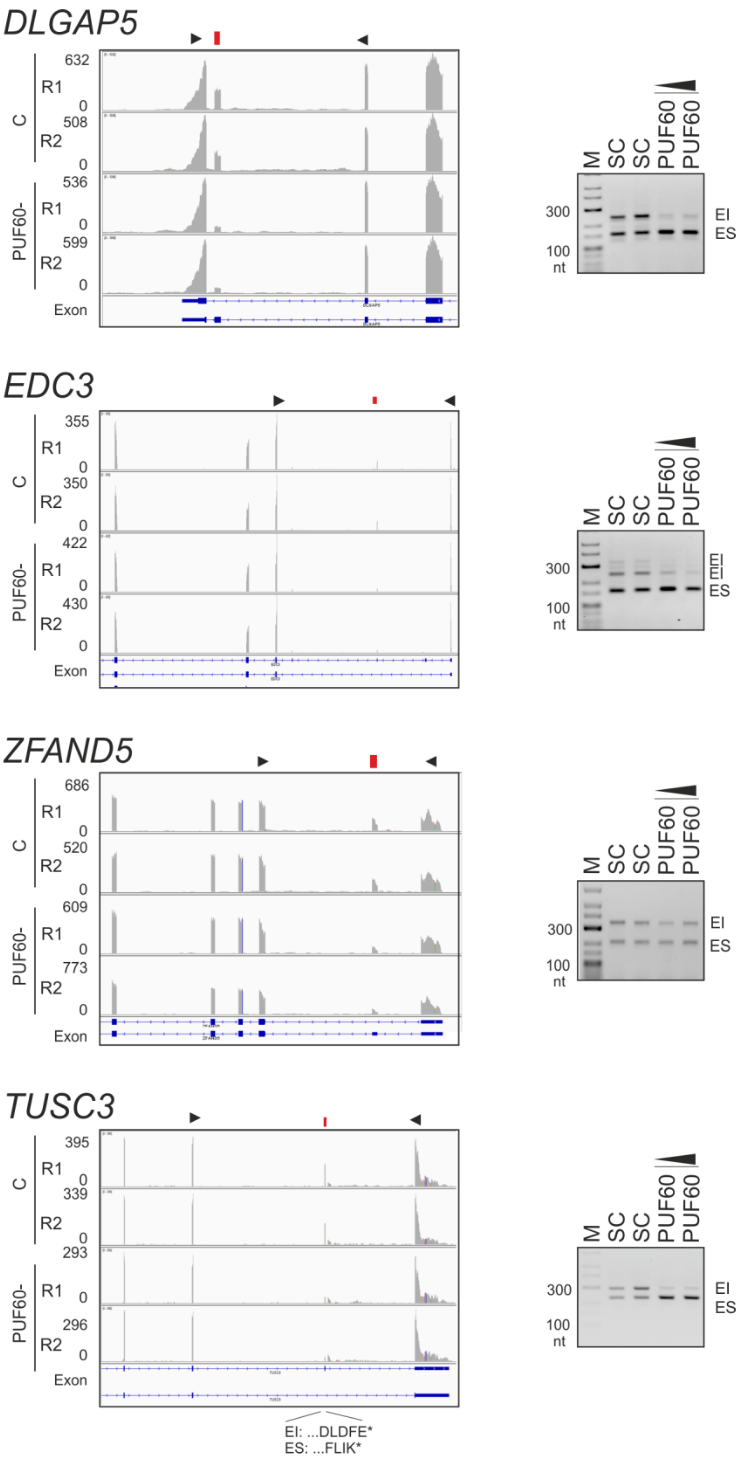
DLGAP5
C
R1
R2
PUF60-
632
508
536
599
0
Exon
M
SC
SC
PUF60
PUF60
300
100
nt
EI
ES
EDC3
355
350
422
430
EI
EI
ES
ZFAND5
686
520
609
773
EI
ES
TUSC3
395
339
293
296
EI
ES
EI: ...DLDFE*
ES: ...FLIK*

C

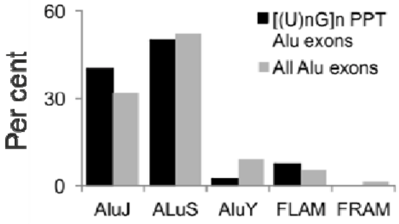
Per cent
60
30
0
[(U)nG]n PPT Alu exons
All Alu exons
AluJ
ALuS
AluY
FLAM
FRAM

D

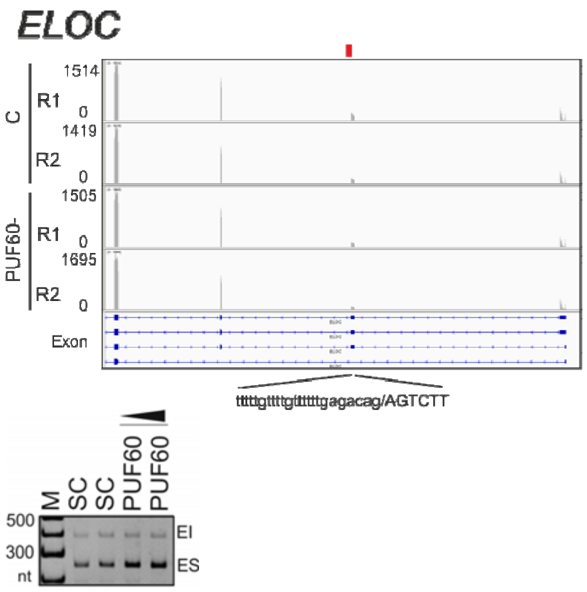
ELOC
C
R1
1514
0
R2
1419
0
PUF60-
R1
1505
0
R2
1695
0
Exon
ttttgttttgtttttgagacag/AGTCTT
M
SC
SC
PUF60
PUF60
500
300
nt
EI
ES

E

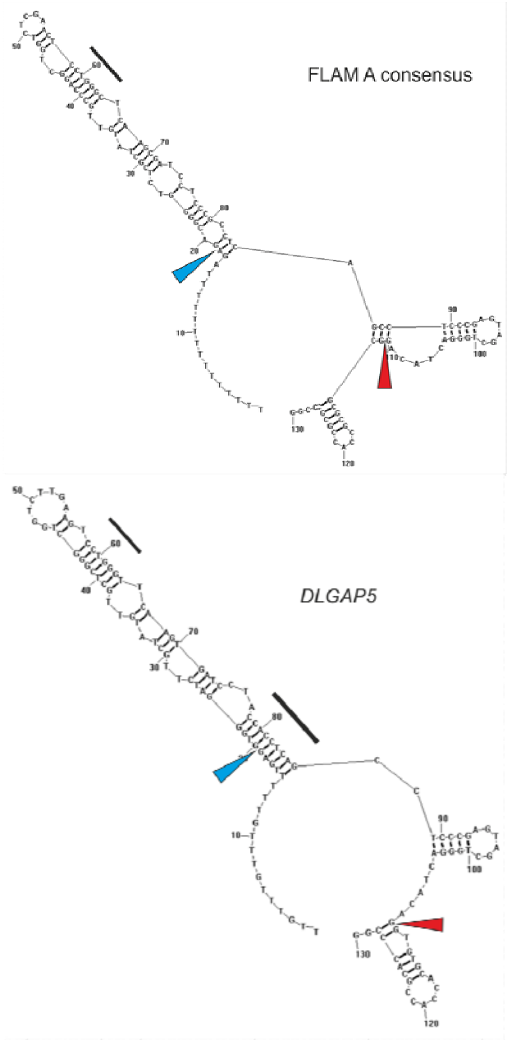
FLAM A consensus
DLGAP5

F

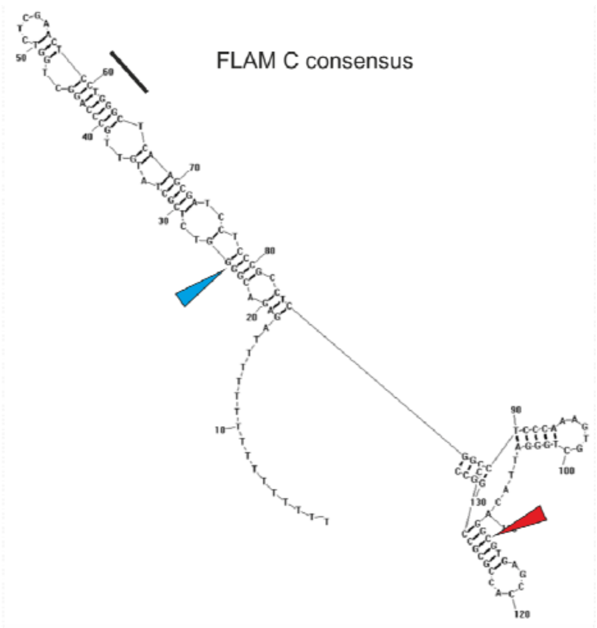

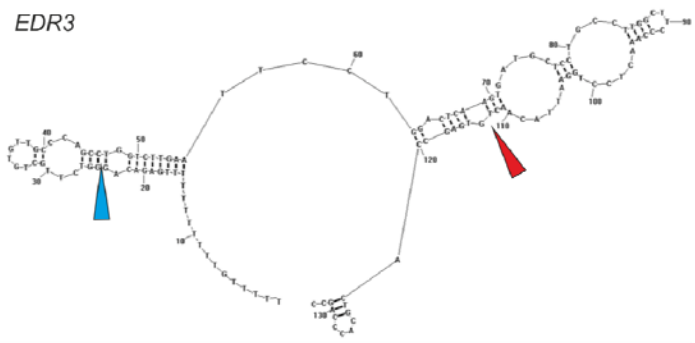

Figure S9 Transposed elements upstream of PUF60-repressed 3’ss

Exons are in upper case and highlighted in grey, introns are in lower case; v and i denote transversions and transitions, respectively. Only the MIR3 element was in the antisense orientation.

```
FAM60A              cacagtgcgctcctgggagatgaagattctggtcatgacctccaacctgg
                                 iv i    i i vvi       i  i  v     i
PRIMA4-int#LT       CACAGTGCGCTCCCTGAAGATAAGGTGCCTGGTCACGATCTGCAACCCGG

FAM60A              aaattttgtctgttggaaaagacatctcataaaggactccctccagcctt
                     i         i               v        i     i  i  ii
PRIMA4-int#LT       AGATTTTGTCTATTGGAAAAGACATCTAATAAAGGATTCCCTTCAACCCC

FAM60A              ggtggaggggcccttaccaggtatcgttgactaatccatgcacagcagag
                     i    i      v         iii              ii v   i i
PRIMA4-int#LT       GATGGAAGGGCCCATACCAGGTACTATTGACTAATCCATGTGCCGCAAAA

FAM60A              ttaaaggttgtagactcacggatccatatctctcaccttaaaaagGCACA
                       i   v i        i    i  i        i
PRIMA4-int#LT       TTAGAGGGTATAGACTCATGGATTCACATCTCTCATCTTAAAAAGGCACA

FAM60A              ACTTCCTGAATGGACCATGACTCCCACCAAAGATCCCTGTCTCTGATTCA
                      i      i     ii i              i iii i   iii
PRIMA4-int#LT       ACCTCCTGAGTGGACTGTAACTCCCACCAAAGACCTTCGCCTCCAGTTCA

FAM60A              CCAAACAGCTTCAACCCTGAAACCAGGACGAGAAGTTGACAACATCTG-A
                     i     ---  i   i v  v      iv     iv   i   v   -v
PRIMA4-int#LT       CTAAACA---TCGACCTTCAACCCAGGATTAGAAGCAGACGACAGCTGTT

FAM60A              GTGGACAGCTAATTGACCTAAGACTTCAGACCAGGCCTgtat
                          v   -- vi   i     vivi
PRIMA4-int#LT       GTGGACTGCT--TAAACCCAAGACACAGGACCAGGCCTGTAT


SNU13               actccaacctaggcgacagagggagactccgtctctaaataaataaa..96nt..ag
                          i   i          v             v   v   v
AluY_short_#S       ACTCCAGCCTGGGCGACAGAGCGAGACTCCGTCTCAAAAAAAAAAAA


FAM161A             gcactccagcctgggcgacagagcgagactccatctcaaaaa..180..nt..ag
                                                    i
AluY_short_#S       GCACTCCAGCCTGGGCGACAGAGCGAGACTCCGTCTCAAAAA


YIF1B               GCCGGGCGCGGTGGCTCATGCTTGTAATCCCAGCACTTTGGGAGGCCGAG
                                      i  i
AluSc8#SINE/A       GCCGGGCGCGGTGGCTCACGCCTGTAATCCCAGCACTTTGGGAGGCCGAG

YIF1B               GCGGTCGGATCACGAGgtcaggagatcaagaccatcctggctaacacggt
                        v                      i
AluSc8#SINE/A       GCGGGCGGATCACGAGGTCAGGAGATCGAGACCATCCTGGCTAACACGGT

YIF1B               gaaaccccatctctactaaaaatacaaaaaattagctgggtgtggtggtg
                            i                           i   i       ii
AluSc8#SINE/A       GAAACCCCGTCTCTACTAAAAATACAAAAAATTAGCCGGGCGTGGTGGCA

YIF1B               tgtgcctgtagtcccagctactcaggaggctgaggcaggagaatcgcttg
                    i i                    i
AluSc8#SINE/A       CGCGCCTGTAGTCCCAGCTACTCGGGAGGCTGAGGCAGGAGAATCGCTTG

YIF1B               aacccaggaggtggaggttgcagtgagccgagatcgcacacctgc
                         i     i                         i vv
AluSc8#SINE/A       AACCCGGGAGGCGGAGGTTGCAGTGAGCCGAGATCGCGCCACTGC


LAS1L               catgttacagATGGTCAGACTGAGGTCCAGAGAGGGGAAGGTACTGACCC
                       v         ivv i       i         v    vv   vi
MIR3#SINE/MIR       CATTTTACAGATGAGGAAACTGAGGCCCAGAGAGGTGAAGTGACTTGCCC

LAS1L               AAAGTCACACAgtgagttattgacaaagctgaagctagaacc-agatctc
                      i         i?     v  i  i     iii        -  i
MIR3#SINE/MIR       AAGGTCACACAGCKAGTTAGTGGCAGAGCTGGGACTAGAACCCAGGTCTC
```

Figure S10 Functional annotation of genes containing PUF60-activated exons

P values are shown to the right of each column.

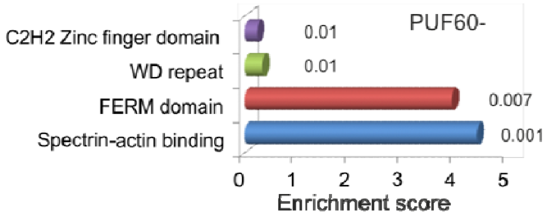

Figure S11   H169 and D159 residues in a PUF60 structure

H169 and D159 are in yellow in a ribbon representation of PUF60 RRM1/RRM1 (Protein DataBank code: 2KXF). H169 is in the exposed loop between β2 and β3 in the vicinity of β3 RNP1 (KGFAFVEY) (*upper panel*) whereas D159 is in β2 (9) (*lower panel*) (Fig. 7J).

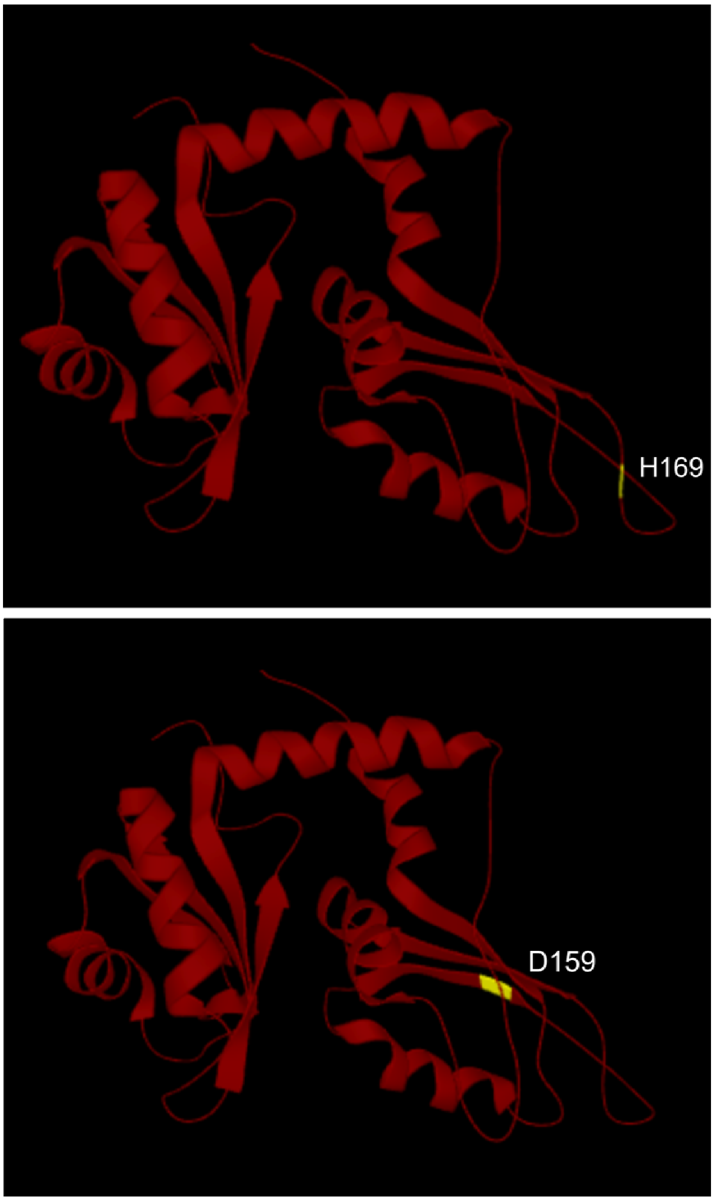

Figure S12 Sequence optimality scores for human PUF60

A, Protein DataBank code: 2QFJ (10). **B,** Protein DataBank code: 5KVY (unpublished). Numbering of amino acids is the same as in PDB entries. The sequence optimality Γ scores (shown below each residue) quantify the degree of non-optimality of amino acids at each amino acid position with respect to the overall protein stability (11). Strongly negative values indicate positions where substitutions can be stabilizing (11). Residues mutated in PD are denoted by arrows. Helices, beta strands and coil regions are in red, blue and green, respectively.

A

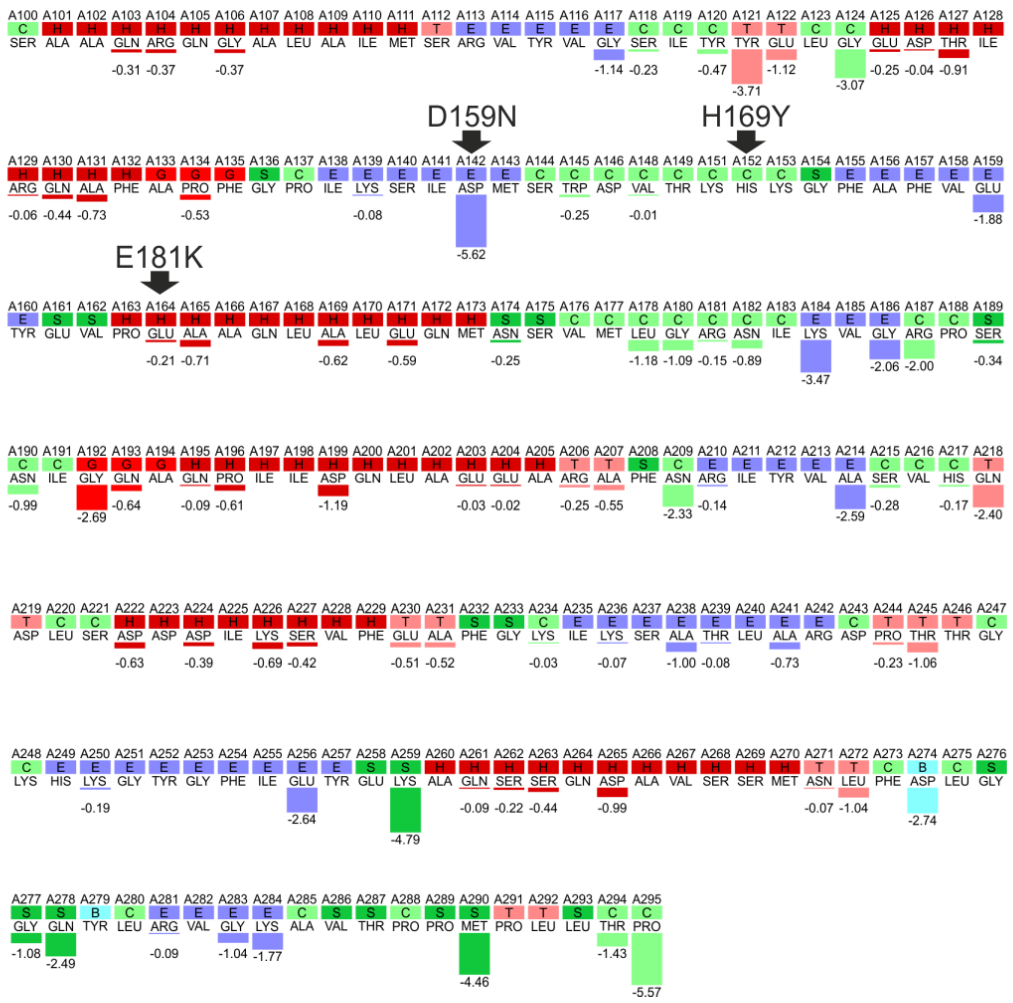

B

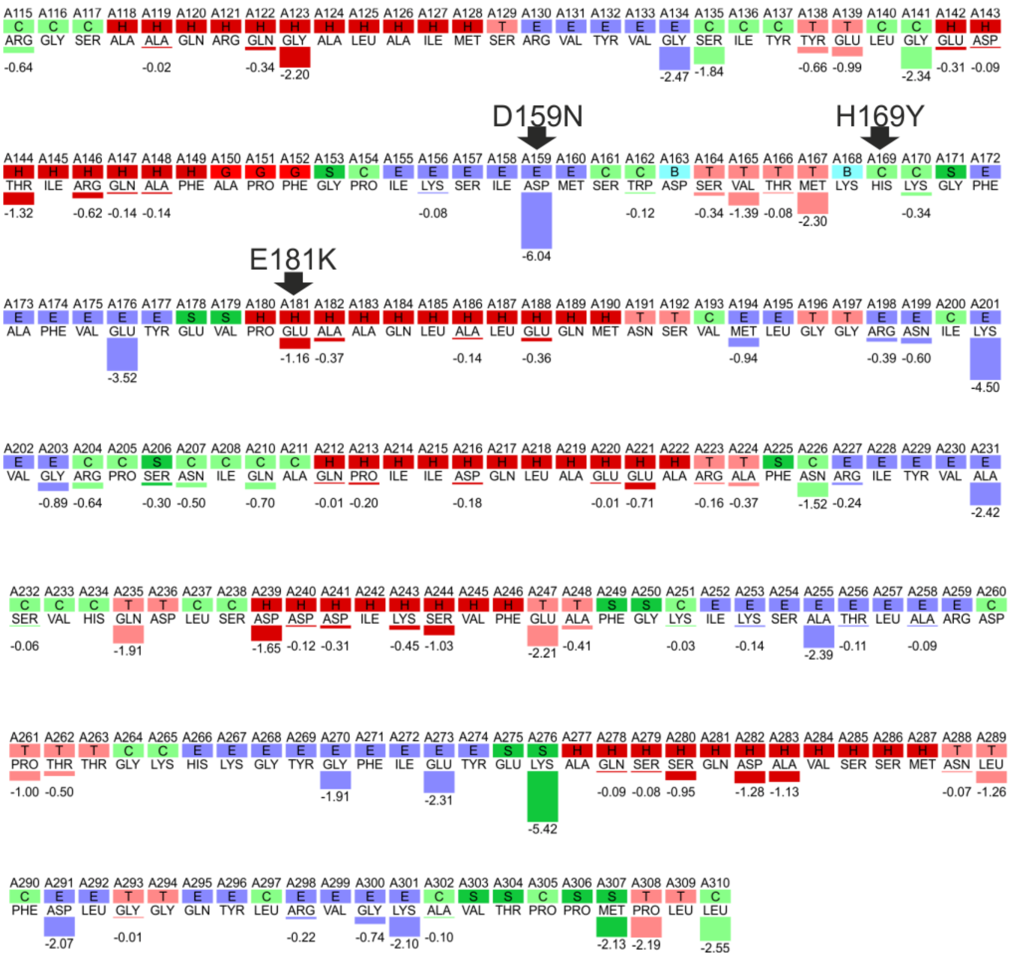

Figure S13 BP mapping of competing *UBE2F* 3’ss in cells overexpressing PUF60

A, Nucleotide sequence upstream of the canonical 3’ss of *UBE2F* exon 5. Predicted BPs are denoted by circles. Shifts in BP/3’ss usage in cells overexpressing PUF60 are schematically depicted by arrows. **B,** BP mapping primers (Table S1). Primers R1 and R2 target intronic sequences derived from *U2AF1* and primers F1 and F2 target *UBE2F*. The minigene is shown in Fig. 7A. The sequence of the 5’ end of the hybrid intron (grey rectangle) is in panel D. **C,** PAGE-separated PCR products amplified by primers F2/R2 using templates shown at the top. F1/R1, first PCR; RT, reverse transcriptase; EV, total RNA from HEK293 cells co-transfected with the *UBE2F* reporter and an empty vector (EV); dATG, total RNA from HEK293 cells co-transfected with the *UBE2F* reporter and the PUF60 dATG construct, which most strongly activated the cryptic 3’ss (lane 9, Fig. 7D,E); C is total RNA from HEK293 cells that were transiently transfected with the *PUF60* minigene (Fig. 7L) as a control. Lanes 6 and 7 contain reamplified plasmid inserts as a size control (sequenced in panel D). Asterisks denote artefacts/heteroduplexes. **D,** Representative chromatograms of two subloned cDNA fragments extracted from lanes 3 and 4.

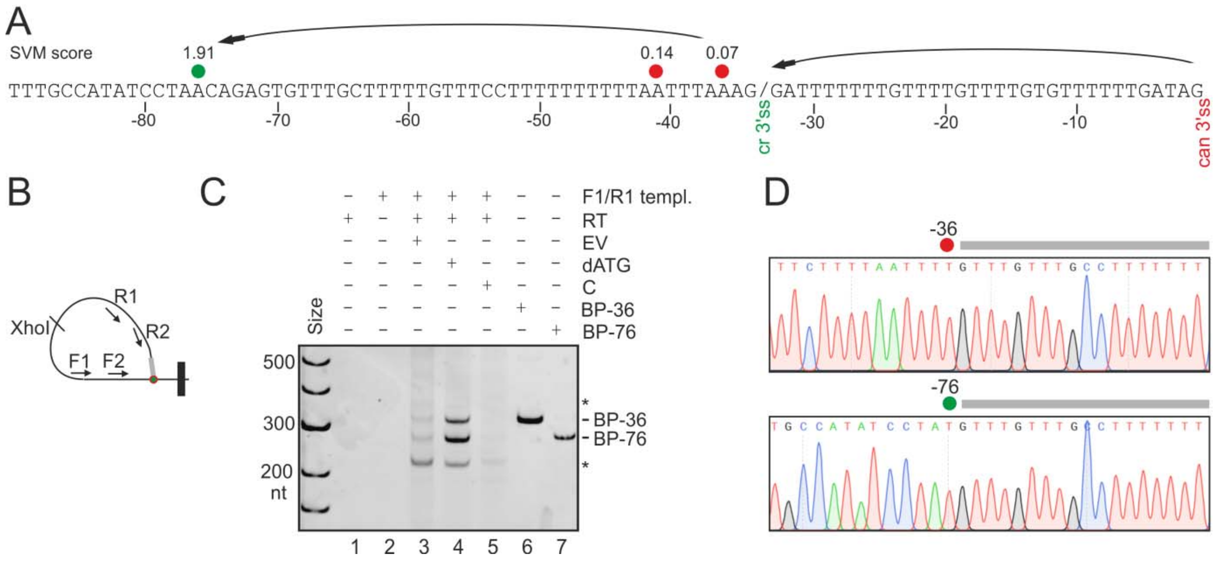

Figure S14 Domain structure of the PUF60-regulated RBP network

The first column gives dinucleotide binding preferences of RBPs shown in the second column. PUF60-regulated transcripts are boxed. Domain structures (aligned to the first RRM) were generated by SMART (12). Low complexity regions are shown as pink rectangles, coil-coiled regions as green rectangles. HuC and HuD were not expressed in our PUF60-depleted HEK293 cells.

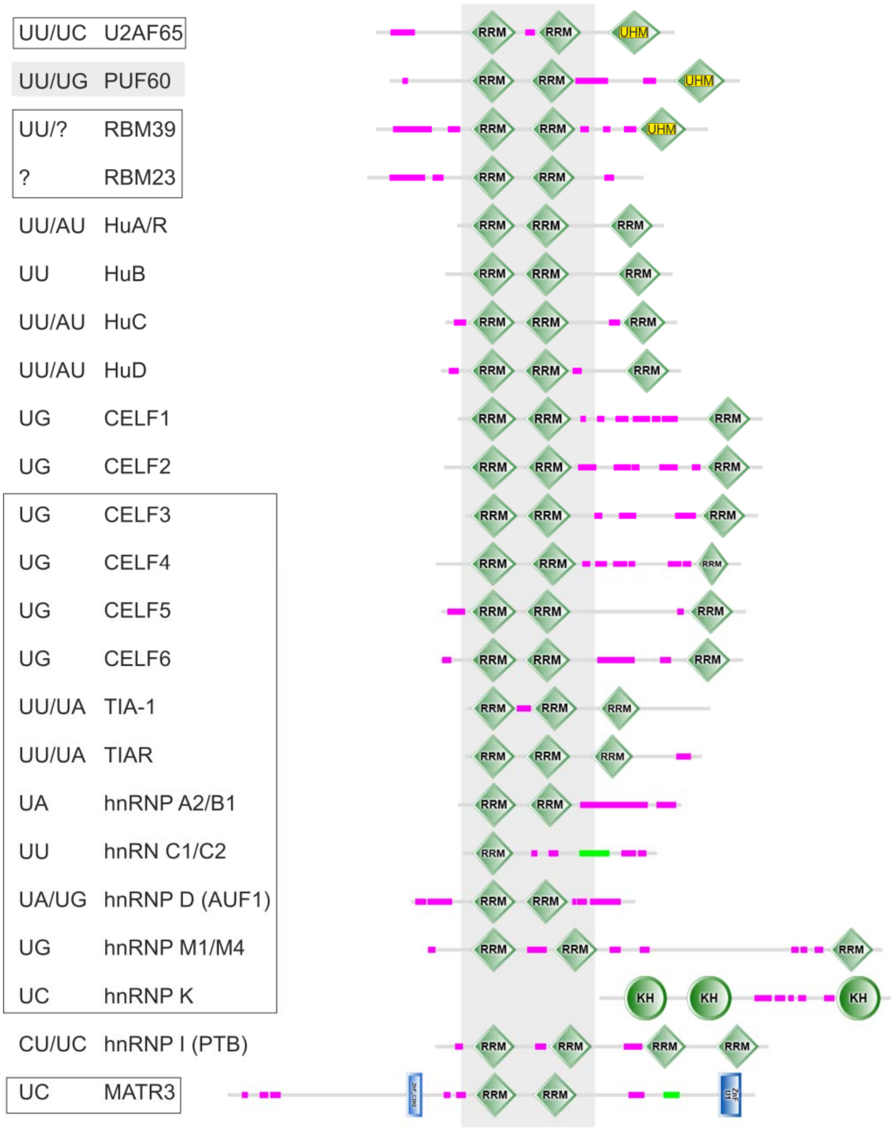

References to Supplemental data

1. Kralovicova, J., Knut, M., Cross, N.C. and Vorechovsky, I. (2015) Identification of U2AF(35)-dependent exons by RNA-Seq reveals a link between 3' splice-site organization and activity of U2AF-related proteins. *Nucleic Acids Res.*, **43**, 3747-3763.
2. Ray, D., Kazan, H., Cook, K.B., Weirauch, M.T., Najafabadi, H.S., Li, X., Gueroussov, S., Albu, M., Zheng, H., Yang, A. *et al.* (2013) A compendium of RNA-binding motifs for decoding gene regulation. *Nature*, **499**, 172-177.
3. Kralovicova, J. and Vorechovsky, I. (2017) Alternative splicing of U2AF1 reveals a shared repression mechanism for duplicated exons. *Nucleic Acids Res.*, **45**, 417-434.
4. Mercer, T.R., Clark, M.B., Andersen, S.B., Brunck, M.E., Haerty, W., Crawford, J., Taft, R.J., Nielsen, L.K., Dinger, M.E. and Mattick, J.S. (2015) Genome-wide discovery of human splicing branchpoints. *Genome Res.*, **25**, 290-303.
5. Baury, B., Masson, D., McDermott, B.M., Jr., Jarry, A., Blottiere, H.M., Blanchardie, P., Laboisse, C.L., Lustenberger, P., Racaniello, V.R. and Denis, M.G. (2003) Identification of secreted CD155 isoforms. *Biochem. Biophys. Res. Commun.*, **309**, 175-182.
6. Bailey, T.L., Boden, M., Buske, F.A., Frith, M., Grant, C.E., Clementi, L., Ren, J., Li, W.W. and Noble, W.S. (2009) MEME SUITE: tools for motif discovery and searching. *Nucleic Acids Res.*, **37**, W202-208.
7. Roca, X., Krainer, A.R. and Eperon, I.C. (2013) Pick one, but be quick: 5' splice sites and the problems of too many choices. *Genes Dev.*, **27**, 129-144.
8. Denton, R.M., Pullen, T.J., Armstrong, C.T., Heesom, K.J. and Rutter, G.A. (2016) Calcium-insensitive splice variants of mammalian E1 subunit of 2-oxoglutarate dehydrogenase complex with tissue-specific patterns of expression. *Biochem. J.*, **473**, 1165-1178.
9. Cukier, C.D., Hollingworth, D., Martin, S.R., Kelly, G., Diaz-Moreno, I. and Ramos, A. (2010) Molecular basis of FIR-mediated c-myc transcriptional control. *Nat. Struct. Mol. Biol.*, **17**, 1058-1064.
10. Crichlow, G.V., Zhou, H., Hsiao, H.H., Frederick, K.B., Debrosse, M., Yang, Y., Folta-Stogniew, E.J., Chung, H.J., Fan, C., De la Cruz, E.M. *et al.* (2008) Dimerization of FIR upon FUSE DNA binding suggests a mechanism of c-myc inhibition. *EMBO J.*, **27**, 277-289.
11. Dehouck, Y., Kwasigroch, J.M., Gilis, D. and Rooman, M. (2011) PoPMuSiC 2.1: a web server for the estimation of protein stability changes upon mutation and sequence optimality. *BMC Bioinformatics*, **12**, 151.
12. Letunic, I., Doerks, T. and Bork, P. (2015) SMART: recent updates, new developments and status in 2015. *Nucleic Acids Res.*, **43**, D257-260.
